# Conductive Biomaterials as Bioactive Wound Dressing for Wound Healing and Skin Tissue Engineering

**DOI:** 10.1007/s40820-021-00751-y

**Published:** 2021-12-02

**Authors:** Rui Yu, Hualei Zhang, Baolin Guo

**Affiliations:** 1 State Key Laboratory for Mechanical Behavior of Materials, and Frontier Institute of Science and Technology, Xi’an Jiaotong University, Xi’an, 710049 People’s Republic of China; 2 Key Laboratory of Shaanxi Province for Craniofacial Precision Medicine Research, College of Stomatology, Xi’an Jiaotong University, Xi’an, 710049 People’s Republic of China

**Keywords:** Conducting polymers, Inorganic nanomaterials, Biomaterials, Electrotherapy, Wound monitoring

## Abstract

Conductive biomaterials based on conductive polymers, carbon nanomaterials, or conductive inorganic nanomaterials demonstrate great potential in wound healing and skin tissue engineering, owing to the similar conductivity to human skin, good antioxidant and antibacterial activities, electrically controlled drug delivery, and photothermal effect. However, a review highlights the design and application of conductive biomaterials for wound healing and skin tissue engineering is lacking. In this review, the design and fabrication methods of conductive biomaterials with various structural forms including film, nanofiber, membrane, hydrogel, sponge, foam, and acellular dermal matrix for applications in wound healing and skin tissue engineering and the corresponding mechanism in promoting the healing process were summarized. The approaches that conductive biomaterials realize their great value in healing wounds via three main strategies (electrotherapy, wound dressing, and wound assessment) were reviewed. The application of conductive biomaterials as wound dressing when facing different wounds including acute wound and chronic wound (infected wound and diabetic wound) and for wound monitoring is discussed in detail. The challenges and perspectives in designing and developing multifunctional conductive biomaterials are proposed as well.

## Highlights

- The design and application of conductive biomaterials for wound healing are comprehensively reviewed, including versatile conductive agents, the various forms of conductive wound dressings, and different in vivo applications.
- Three main strategies of which conductive biomaterials realizing their applications in wound healing and skin tissue engineering are discussed.
- The challenges and perspectives in designing multifunctional conductive biomaterials and further clinical translation are proposed.

## Graphical abstract

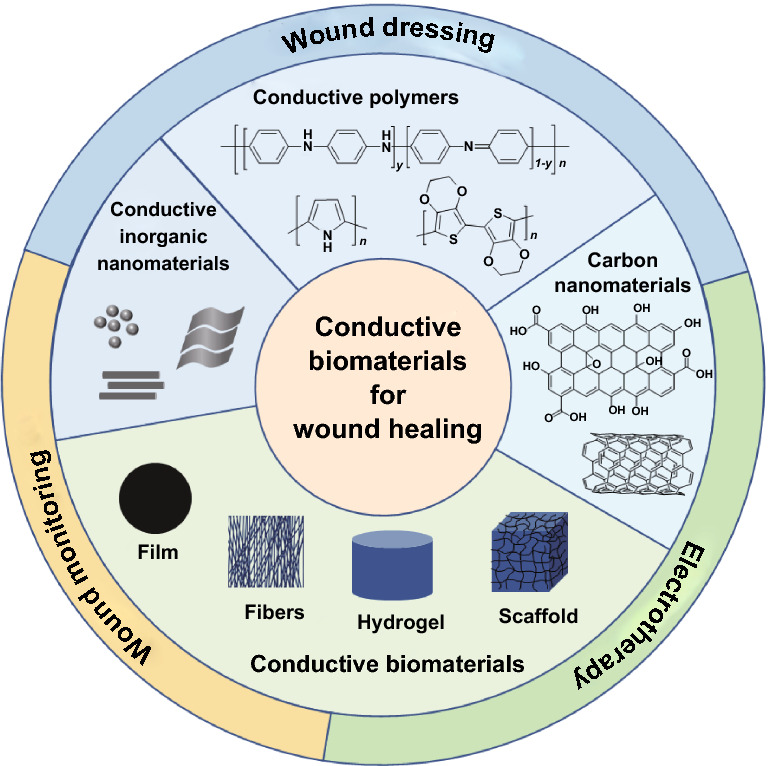

## Introduction

As the largest organ of the human body, skin displays significant influence on diverse human activities and functions, including protection from pathogens, sensing of external environment, and thermoregulation [1]. However, laying on the outmost of the human body and facing incessant conflicts, skin owing to its elastic and soft nature is susceptible to generate defects that are referred to as wounds [2, 3]. Even though human skin could self-repair spontaneously to restore its structural and functional integrity, wound care is still of great necessity to prevent infection and desiccation, alleviate pain, protect the open site, accelerate the healing process, and avoid scar formation, especially for large and open wounds or burns [4–6]. In 2014, 17.2 million hospital visits by acute wounds were recorded in the USA [7]. Currently, about 1–2% of the population in developed countries suffers from chronic wounds [8]. Meanwhile, ascribing to diseases, aging or inappropriate treatment, chronic wounds as diabetic ulcers, vascular ulcers, and pressure ulcers possessing a long and cruel healing period do not only affect patients’ daily life but also associate with high morbidity and mortality [9, 10]. The global advanced wound care products market is growing rapidly, about $12 billion in 2020 and estimated to be $18.7 billion by 2027 [7]. At present, wound healing remains a hot and challenging issue both in clinic and scientific research.

To date, the physiology of wound healing has been well established [11]. The healing process comprises four overlapping phases: hemostasis, inflammation, proliferation, and remodeling, along with diverse growth factors, enzymes, and cytokines exerting significant effects in synergistically modulating relevant cell activities [5, 12–14]. There are many types of wounds: acute incision and excision wound can go through normal healing process, while chronic wounds have aberrant healing conditions [10]. In clinic, wound healing management varies according to the tissue feature, the intrinsic regenerative capacity, wound classification, and other environmental variables [6, 10, 15–17]. The therapeutic strategies for wounds are versatile, including hyperbaric oxygen therapy [18–20], negative-pressure therapy [21], vacuum-assisted closure [22], ultrasound [23], electrotherapy [24, 25], auto/allograft and xenograft [4, 26, 27], cell-based therapy and engineered skin graft [4, 28, 29], and topical drug and growth factor delivery [30–32]. No matter which class the wounds belong to, and which wound care strategy would be chosen, wound dressing is requisite [33]. Traditional passive wound dressings like gauze, bandage, and cotton wool could hardly fit the open wounds and exert no active effect in wound healing [9]. Even worse, they would adhere to the skin tissue, causing dehydration and second injury upon replacement. In contrast, modern biomaterial-based wound dressings integrating multifunction as maintaining a moist environment, managing exudate and protection from pathogens, antibacterial capacity, antioxidant property, injectability, self-healing capacity, adhesiveness, and suitable mechanical properties have recently surged and demonstrated extraordinary advantages in more complicated situations [4, 34, 35].

Except for the above-mentioned features, our human skin possesses another key characteristic, the conductive nature of the intact skin, which plays a vital role in human activities [36–38]. Once the skin is disrupted, endogenous wound-induced electric fields ranging from 40–200 mV/mm generate and immediately initiate wound healing [39–42]. Accordingly, electrical stimulation-based electrotherapy has been developed and applied in practice since the end of the twentieth century, especially for chronic wounds. The electrical stimulation (ES) accelerates the wound healing process in all stages through diverse pathways [39–41, 43–45]. It can alleviate edema around the electrode, guide keratinocyte migration, enhance re-epithelialization, direct dermal angiogenesis, modulate a variety of genes relevant with wound healing, and generate antibacterial effects [43, 46, 47]. But the application of electrotherapy must be carried out with a large extracorporeal ES device and requires careful and precise evaluation of the relevant parameters, including voltage, current, mode, and working time, depending on the nature and condition of the wound [48, 49]. Moreover, the efficacy of electrotherapy is limited by the uneven distribution of ES ascribing to a large amount of body fluid, irregular wound shape, the exudates induced metal electrodes corrosion, and wound status [24, 25, 50].

In the past decade, wound treatment strategy relating to the conductive nature of human skin has emerged and attracted much attention for its high efficacy, processing flexibility, and ease in handling and management. The sophisticated conductive biomaterial-based wound dressings with similar conductivity to that of human skin have demonstrated significant enhancement in wound healing and exhibited great potential in different types of wounds, as full-thickness acute wound, infected wound, and diabetic wound [37, 51, 52]. The basic principle of fabricating conductive wound dressing is to incorporate the electroactive substances mainly including carbon nanomaterial [5, 53, 54], conductive polymers (CPs) [55, 56], or metal-based materials [57–60] into the polymeric biomaterial [51]. So far, there have designed a lot of conductive wound dressings in different forms, as film, membrane, electrospun nanofiber, hydrogel, cryogel, and foam [37, 61, 62]. Besides, since the advanced development of tissue engineering and regenerative medicine, conductive scaffolds mimicking ECM loaded with bioactive molecules or cells have also been delicately developed for more severe wounds as large or open wounds and chronic wounds with poor regenerative abilities, providing mechanical support and modulating cell activities [63, 64]. Usually, sponge and foam, hydrogel, and nanofibrous network with highly porous structures are ideal for scaffolds [65]. However, a comprehensive review summarizing the design and application of conducive biomaterials for wound healing and skin tissue engineering is still lacking.

**Fig. 1:**
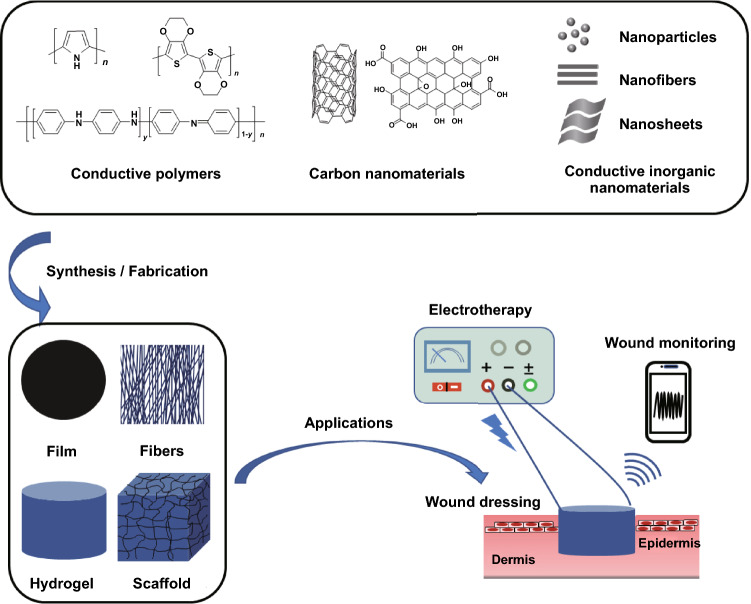
Schematic illustration of conductive biomaterials in wound healing and skin tissue engineering, including fabrication, forms, and applications

This review article comprehensively summarizes the recent immense achievement and great potential of the conductive wound dressings for wound healing and skin tissue engineering. The advantages and drawbacks of different conductive substances are summarized (Fig. 1). The current fabrication methods of conductive biomaterial in different forms are reviewed and classified by 2D and 3D morphologies (Fig. 1), as well as their applications in different wound models and the related aspects to consider when facing specific wound were deliberated. Three main strategies of conductive biomaterials in promoting wound healing were summarized. The mechanism of conductive materials on accelerating wound healing is also discussed. Commonly, conductive materials do not work alone, but are synergistically utilized with other functional materials, thereby the combination are also recorded, and the synergistic effects are discussed (Fig. 1). Furthermore, considering the current achievements and clinical needs, we envision the future directions and propose the challenges of conductive biomaterials in wound healing and skin tissue engineering.

## General Design Principles of Conductive Biomaterials for Wound Healing

Three main issues need to be carefully evaluated when designing conductive biomaterials for wound healing. First is the selection of matrix material. Naturally derived polymers have good biocompatibility, enzymatic or hydrolytic reactions-based biodegradability, and versatile biological properties which could promote wound healing, but their quality varies from batch to batch [56, 66]. In contrast, synthetic polymers have more controlled structure and superior mechanical properties, but poor cell attachment owing to their hydrophilicity [67]. Biocompatibility and degradability are other two critical issues that impair the biomedical applications of synthetic polymers [68, 69]. Besides, they usually lack bioactivity and need to be functionalized to meet biological requirements. Therefore, in numerous cases, natural and synthetic polymers are combined either through simple blending or crosslinking to integrate multifunction, including biocompatibility, degradability, and specific bioactivity and suitable mechanical strength. So far, various natural and synthetic polymeric materials have been utilized to fabricate wound dressings, including chitosan, fibrin, gelatin, hyaluronic acid, alginate, cellulose, silk fibroin, polyethylene glycol, polycaprolactone, poly(lactic acid), poly(glycolic acid), polyvinyl alcohol, and polyurethane [37, 70, 71]. These biomaterials exhibit diverse properties due to the intrinsic unique features of the components and the fabrication methods.

Except for the composition, the structural morphology is another key factor for biomaterials that notably determines the fabrication method, properties, and performance [9]. Commonly, film, membrane, and fibers possessing 2D structure have good oxygen permeability, resistance toward water and tough mechanical properties. Sponges, foam, and hydrogel owning 3D network and porous structure could absorb large amount of exudate, maintain moist environment, and act as carriers for bioactive substances and cells [72, 73]. The applications of these biomaterials also depend on their morphology. 2D biomaterials are usually used as wound dressing, while those with 3D structure can be fabricated as wound dressings and tissue scaffolds. Particularly, as nanofibers could be programmed into ECM-liked aligned architecture, they also have great potential in skin scaffolds [26]. Meanwhile, the application of these conductive biomaterials is somewhat limited by their morphology. 2D biomaterials cannot adapt to deep and chronic wounds, and foams with tough nature could not be applied on delicate skin and dry wound, while hydrogel with relative soft nature may cause wound dehydration and fail long-term use due to inevitable water evaporation and the constant movement of the wounds [9]. It is worth noting that in the cases of the combination of natural and synthetic polymers, the structural morphology of these biomaterials also affects the selection of fabrication methods. Specifically, simple blending could be found in fabricating 2D biomaterials [74]. Chemical or physical crosslinking is an alternative utilized to further enhance the stability and mechanical properties. Meanwhile, crosslinking is essential when designing 3D biomaterials, where permanent covalent bonds usually result in a tough matrix, while physical crosslinking and dynamic covalent bonds usually result in soft biomaterials.

Third comes to the incorporation of conductive materials. So far, several types of conductive materials have been elaborately studied and explored. They can be well-tuned and controlled into different morphologies, as nanoparticles, nanowires, nanotubes, and nanosheets, which has a significant impact on their properties [53, 75, 76]. The excellent electroconductivity, unique optical properties, and relevant antibacterial and photothermal properties have led to extensive applications of conductive materials in diverse fields, such as sensors, conductors, supercapacitors, medical, tissue engineering, energy storage, and so on [51, 77–81]. However, the incorporation of the conductive substances into biomaterials remains a great challenge. Most CPs, carbon nanomaterials, metals, and metal oxides are insoluble in aqueous solution and tend to aggregate resulting in a lower conductivity [82–85]. Thus, the homogeneous dispersion of these conductive substances requires elaborate pretreatment. Surface modification is a mature method to improve the solubility and stability of the conductive nanomaterials [86]. Hydrophilic molecules including small molecules and polymers can be grafted onto conductive nanomaterials through chemical conjugation or noncovalent interactions. Polymer wrapping assisted by strong stirring and sonication has been proved effective in promoting the even dispersion of nanosized conductive materials and preventing aggregation. Besides, functionalization of conductive polymers could be realized through doping mechanism [52]. The stability, degradability, and cytotoxicity of CPs under physiological conditions also matter a lot [87]. Carbon nanomaterials and metal nanomaterials could not even degrade in vivo [88]. Besides, the incorporation of conductive substances would make a great difference on the mechanical properties of the pristine biomaterial [89]. The balance and compromise between the conductivity, biocompatibility and mechanical properties should be fully investigated before in vivo application.

Excitingly, novel 2D inorganic conductive nanomaterials as black phosphorus (BP) and transition metal carbides and nitrides (MXene) have recently attracted great attention for their electroactivity and demonstrated great potential in biomedical applications for the biodegradability, photothermal effect, and antibacterial activities [90–94]. On the other hand, their applications are hindered by poor stability under ambient conditions or in liquid medium [81, 95]. The applications of BP and MXene in wound healing are still in the very preliminary stage while facing plenty of challenges and new opportunities. In this context, these conductive biomaterials are reviewed in two categories, 2D and 3D conductive biomaterials. In addition, we focus on providing brief guide to the fabrication methodology for incorporating conductive materials into wound dressings and scaffolds.

## 2D Conductive Biomaterials for Wound Healing

### Film

Thin film is semipermeable, allowing oxygen and water vapor to pass through, while a large amount of fluids and bacteria are blocked. Due to the flexibility, lightweight, and poor water-uptake capacity, film-based wound dressing is suitable for delicate skin and thin wounds with little exudate [96]. Meanwhile, the film should not be too compact, because low vapor transmission and oxygen permeability would deteriorate wounds resulting from moisture accumulation and skin maceration. Thin films can also serve as matrices for bioactive reagents, including conductive materials. Due to ease handling both for in vitro and in vivo, conductive thin films have been widely employed to study the mechanism of how conductive materials modulating cell activities, which also facilitates their further applications in other more sophisticated structures [97]. Thus, conductive film-based wound dressings have attracted great attention, and many studies focused on the mechanism of conductive substances promoting wound healing. Since cells were cultured on the surfaces of these films, the vapor transmission and oxygen permeability of these films were not taken into consideration [98–100].

Films are usually fabricated via a solvent-casting method [97, 100, 101]. Thus, compared with carbon nanomaterials that tend to aggregate and need assistance from other amphipathic polymers or technology to form monodispersion, CPs synthesized via polymerization through solvent-soluble monomers demonstrate tremendous advantages in designing conductive films [102, 103]. Table 1 lists previously reported conductive films as wound dressings.

**Table 1:** Conductive films for wound healing applications

Components	Fabrication method	Conductivity	Electrical stimuli	Cell type	Animal model	Refs.
PPy/PSS/HA	Chemical polymerization	8.02 S cm^−1^	N/A	Rat PC-12 cells	N/A	[104]
PPy/Cl, PVS, Hep, Derm, Fbri, Fn, Col	Electrochemical polymerization	N/A	N/A	SVK14 keratinocyte, primary keratinocytes	N/A	[98]
PEDOT	Electrochemical polymerization	N/A	N/A	Epithelial cells	N/A	[99]
PPy	Chemical polymerization, casting	10^–3^ S cm^−1^	Direct field strength 100 mV mm^−1^	Human skin fibroblasts	N/A	[101]
PEDOT:Tosylate	Chemical polymerization, spin coating, bar coating	N/A	1.0 V, 1.5 V/24 h	Madin Darby canine kidney cells	N/A	[105]
PPy/PLLA/Fn/BSA	Chemical polymerization, Casting	10^–1 ^S cm^−1^	N/A	Human skin fibroblasts	N/A	[106]
PEDOT:Tosylate	Vapor phase polymerization	N/A	− 1 − + 1 V	Mouse fibroblasts 3T3-L1, MDA-MB-231	N/A	[107]
PEDOT:Tosylate	Vapor phase polymerization	N/A	− 1 − + 1 V	Bovine aortic endothelial cell	N/A	[108]
PEDOT:Tosylate/Glycol	Vapor phase polymerization	1500 S cm^−1^	± 100 mV, ± 300 mV	Mouse fibroblast human keratinocyte cell	N/A	[109]
Graphene/CS	EDC/NHS	0.3 S m^−1^	N/A	N/A	N/A	[110]
Zn/Ag/Polyester	Pattern printing	0.2 V, 1 V between silver and zinc dots	N/A	Human keratinocytes	N/A	[111]
GO/Col/Fibrin	Casting	N/A	N/A	NIH 3T3 fibroblast cell	I	[100]
APPy	Electrochemical polymerization	3.1 × 10^–3^ -24 S cm^−1^	N/A	Human dermal fibroblasts, Schwann cells	N/A	[103]
PEDOT:PSS	Spin coating, electrochemical polymerization	0.36 kΩ sq^−1^	N/A	Primary human dermal fibroblasts, human glioblastoma multiforme cells	N/A	[112]
Zn/Ag/Polyester	Pattern printing	N/A	626 ± 86.3 mV	N/A	N/A	[45]
PPy/Ag NPs/RC/IL	Casting	N/A	N/A	N/A	N/A	[113]
PANI/PHBV/Curcumin	Casting, Schiff base	5.78 × 10^–5^ S cm^−1^	N/A	NIH 3T3 fibroblast cell	N/A	[114]
ZnO NR/PDMS	Spin coating	N/A	320 mV, 900 mV	Human dermal fibroblasts,	I	[115]
Cu/PTFE/PET	N/A	N/A	N/A	NIH 3T3 fibroblast cell	I	[116]
rGO/SA/Gel/HA	Casting	1.19 × 10^–5^ S cm^−1^	N/A	Mouse fibroblasts	N/A	[97]
rGO/Ag NPs/Cellulose	Casting, in situ reduction	N/A	N/A	NIH 3T3 fibroblast cell, A549 human lung epithelial cell	N/A	[117]
Aniline trimer/PCL-PEG	Casting	N/A	N/A	Mouse fibroblasts	I	[118]
PEDOT/Zn^2+^, Cu^2+^/Cellulose	Electrochemical polymerization	N/A	N/A	Human keratinocytes	N/A	[119]
Zn/Ag/Cotton	Magnetron sputtering	N/A	786 mV, 47 μA	Human embryonic lung fibroblasts	I	[120]
rGO/Ag NPs /Cellulose/PDA	Biological blending self-growth method, in situ reduction	0.5062 Ω	N/A	NIH 3T3 fibroblast cell	N/A	[121]
BP/SF	Casting	N/A	N/A	Human synovial fibroblast cells	II	[122]
am-ZnO@CuO@Au/PVA	Casting	N/A	N/A	NIH 3T3 fibroblast cell	III	[123]
CNT/PVPI	Casting	10 kΩ sq^−1^	N/A	N/A	N/A	[124]
CNT/PVPI	Casting	10 kΩ sq^−1^	N/A	Human keratinocytes	N/A	[125]

I: Full-thickness wound. II: E. coli-infected full-thickness wound. III: Full-thickness diabetic wound

Polypyrrole (PPy), poly(3,4-ethylenedioxythiophene) (PEDOT), and polyaniline (PANI) and its oligomers are mostly used for their biocompatibility, facile synthesis, and tunable conductivity generated by various dopants [52, 82, 102]. Even though the pure CP films could be fabricated through electrochemical polymerization with controlled morphology, the inherent brittleness, mechanical rigidity, and nondegradability restrict their applications as wound dressings [79]. Simple blending or conjugation with other elastic biocompatible polymers is a general principle to avoid these limitations [26, 37, 61].

#### PPy

Companying with the surging of ES enhanced wound healing, conductive films have been used as electrodes for the ES devices. PPy-based polymer films have been detailedly explored. Shi group have synthesized PPy nanoparticles via a water-in-oil emulsion system and incorporated PPy into PLLA [101]. This conductive composite film could support cell attachment, spreading, and proliferation of human cutaneous fibroblasts with or without ES. Besides, cell viability was much higher when the composite film was under direct electrical field strength of 100 mV mm^−1^. Later, they explored the detailed mechanism of PPy/PLLA film prompting wound healing. Under ES mediation (100 mV mm^−1^), the conductive PPy/PLLA film could enhance the viability of human cutaneous fibroblasts by modulating cytokines as IL-6 and IL-8 [126]. Mahmoud et al. have fabricated heparin-doped PPy/PLA membrane [127]. When exposed to ES, the conductive film revealed enhanced dermal fibroblast activity with higher expression of FGF-1 and FGF-2 and promoted myofibroblast transdifferentiation with higher level of α-smooth muscle actin. Later, they have proved at genetic level that PPy conductive composite film could modulate the expression of various genes in the wound healing process [128]. In addition, PPy is responsive to electrical signals, so it can be used to design electronically controlled drug delivery [129]. Justin et al. have developed an electronically controlled drug delivery system based on PPy contained conductive polymer film promoting wound healing process [130].

Without ES, PPy-based conductive composite films also have proven positive effects on wound healing. Many studies have proved that the addition of PPy would affect the performance of the conductive films in promoting wound healing to a large extent. Collier et al. fabricated a bilayer film based on PPy doped with PSS as a thin bottom layer and PPy doped with hyaluronic acid as a thick top layer [104]. This bilayer conductive film exhibited superior electrical conductivity, electrochemical properties, surface morphology, and mechanical integrity than single layer only containing HA and PPy. As this bilayer conductive films could promote angiogenesis, the author suggested this bilayer conductive film could act as promising candidate for wound healing application.

Different dopants, including proteins and polysaccharides, can be used to fabricate PPy-contained films [98]. Among them, the film that contained dermatan sulfate revealed the largest effect on supporting keratinocyte growth. PPy nanoparticle could also be modified with fibronectin or bovine serum albumin and then deposited with PLLA to develop bioactivating PPy conductive membrane [106]. The fibronectin-modified PPy/PLLA film supported a higher adhesion and spreading of human skin fibroblasts compared with BSA-modified PPy/PLLA membrane. This was because these incorporated bioactive molecules also play a significant role in modulating cell activities.

The hydrophilicity of film surface is another key factor that affects cellular activities [96]. The introduction of PPy would increase the hydrophobicity of the designed composites, thus generating a negative effect on cell attachment. Amine-functionalized PPy has been proved with improved hydrophilicity compared with pure PPy and demonstrated enhanced adhesion toward human fibroblasts and Schwann cells [103].

#### PEDOT

PEDOT possessing a better thermal stability and higher conductivity than PPy has also been chosen to fabricate conductive films. Firstly, the enhanced cell adhesion and proliferation of epithelial cells on PEDOT films have been convinced [99]. Besides, this PEDOT film showed a remarkable affinity toward protein as bovine serum albumin, thus demonstrating good electrocompatibility and electroactivity. The conductivity of PEDOT can be further enhanced by the introduction of dopant. Wan et al. developed a styrenesulfonate doped PEDOT conductive film named as PEDOT-TOS and combined this film with indium tin oxide assembling a redox gradient along this PEDOT-TOS stripe [107]. The PEDOT-TOS validated cell viability exceeding 98% regardless of its redox states. Besides, under gradient distributed potential, the cultured mouse fibroblasts exhibited density gradient as well, which may associate with differences in orientation of the adsorbed protein at the two electrodes or a higher concentration of adsorbed protein on the reduced side of the device would suppress cell adhesion. The cell density and distribution could be controlled by the dimensions of the conducting film and programmed applied bias. Moreover, this device could be utilized to control cell migration under electrical cues. The migration speed, direction, and directional persistence time of bovine aortic endothelial cells can be enhanced and controlled by the applied bias, thereby indicating the potential of PEDOT-TOS film as wound dressing [108].

**Fig. 2:**
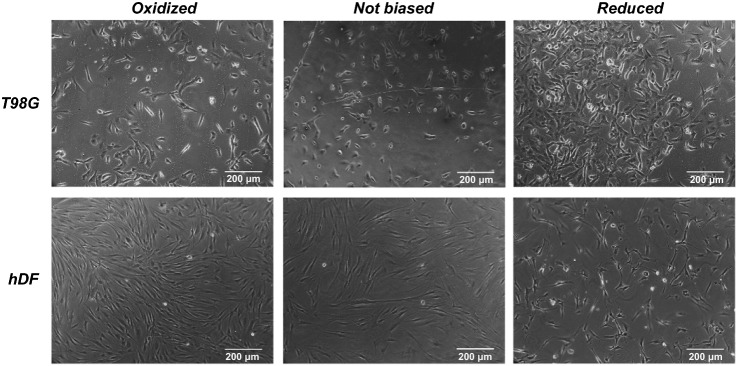
Representative image of T98G and hDF proliferation on PEDOT:PSS based films under different redox states after culturing for 72 h. Reprinted from Ref. [112]. Copyright 2015, American Chemical Society

The polyelectrolyte, PSS, could not only extremely enhance the conductivity of the PEDOT system, but also result in a water-soluble polyelectrolyte system with good film-forming benefit. Marzocchi et al. have managed to deposit PEDOT:PSS on commercial film via spin coating and electrochemical polymerization and proved the films in oxidized state exhibiting enhanced proliferation rate toward hDF [112]. Meanwhile, the enhance cell growth was cell-dependent for that the enhanced growth of T98G was found on reduced substrate, as shown in Fig. 2.

Moreover, Nguyen et al. proved a polycarbonate membrane composed of PEDOT nanotubes could be utilized as a carrier for controlled ex vivo transdermal drug delivery [131]. Under programmed potential, the drug release prolife of insulin could be finely tuned, attributing to the electroresponsive dynamic electrostatic interaction between drugs and PPy nanotube. In summary, PEDOT-based films possess multiple advantages for wound healing application.

#### PANI and Aniline Oligomers

**Fig. 3:**
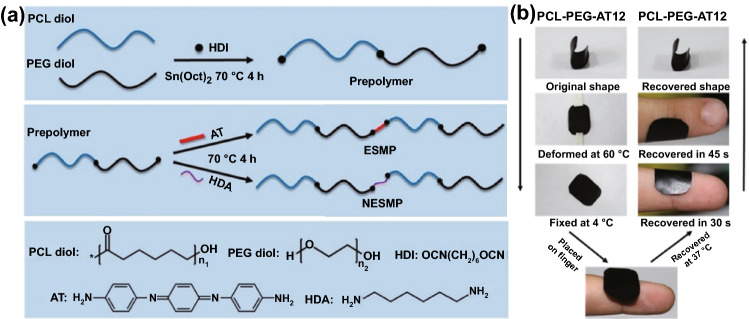
Synthetic procedure of PCL-PEG-AT elastomer (**a**) and its shape memory behavior displaying in practical application (**b**). Reprinted from Ref. [118]. Copyright 2020, Elsevier

Although being one of the most widely used CPs and possessing good chemical and mechanical stability, there are rare reports about pure PANI-based conductive films as wound dressing, probably due to its poor solubility and high infusibility. It is hard to dissolve PANI under mild conditions and further form a thin film [132]. On the other hand, the polymerization of aniline monomers requires an acidic condition (pH < 4) which may be severe to other biomolecules and restrict its direct implementation [82]. To circumvent this issue, Pramanik et al. developed a facile strategy to conjugate PANI nanofibers on the aminated PHBV surface (PHBV-g-PANI) in a methylsufinyl carbanion-treated DMSO solution [114]. Through π-π interactions, curcumin could be entrapped by PANI; thus, this hydrophobic drug was loaded within PHBV-g-PANI. This curcumin-loaded conductive film composite exhibited antibacterial activities and could promote cell migration and proliferation on the injured tissues. However, PANI still exert cytotoxic effect on cells and could not degrade in vivo. In stark contrast, aniline oligomers with well-defined structure, good solubility, biocompatibility, and similar electroactivity have been used to fabricate conductive films in biomedicine and tissue engineering [51]. Our group designed a series of conductive polyurethane–urea elastomer films composed of PCL segment, PEG segment, and aniline trimer [118]. As shown in Fig. 3a, this elastomer adopted a delicate design strategy: the PCL segment provided good mechanical properties, the PEG segment endowed the film with appropriate hydrophilicity and swelling ratio, and aniline trimer ensured electroactivity and antioxidant activity. More impressively, this polyurethane-urea film exhibited shape memory properties (Fig. 3b). Therefore, this film could be manufactured into specific shape suitable for wounds in cylindrical body. When the film in a temporary shape was applied on the wound site, it would rapidly restore to the original shape, thereby generating a force which could facilitate wound closure. In a full-thickness skin wound assay, this conductive elastomer film demonstrated significantly enhanced wound healing performance with the highest collagen deposition and granulation tissue thickness than no-electroactive film and commercial dressing (Tegaderm™). To this end, we suggested that the shape memory properties should be emphasized in wound healing applications as well as its well-established application in minimally invasive surgery, because it could maintain the original shape independent of external force, thereby promoting wound contraction during the initial stage of wound healing.

#### Carbon Nanomaterials

Carbon nanotube (CNT) in single-walled and multi-walled forms has been comprehensively studied and applied in various fields. It is well established that CNT has large specific surface area, electrical conductivity, good mechanical properties, and spectroscopic properties. CNT also demonstrates promising properties in skin tissue engineering, including antibacterial activities, high drug loading efficacy, angiogenesis enhancement, and tissue repair-related gene expression manipulation [85, 133]. However, the cytotoxicity of CNT needs to be fully evaluated when facing the application of direct contact to human cells, considering the current reports that the toxicity of CNT depends on various parameters, including length, diameter, impurities, and modified agents [54, 85].

Although the incorporation of CNT into polymeric matrix endows the composites with enhanced mechanical and physiochemical properties, the incorporation process suffers from the strong tendency to agglomerate and hydrophobic nature of CNT. Polymer wrapping technique is a classical effective method to solubilize CNT [134]. Simmons and his colleagues reported an antiseptic film based on CNT and polyvinylpyrrolidone-iodine, in which CNT was solubilized by polyvinylpyrrolidone-iodine and encased in a polymer monolayer with a helical coil conformation [124, 125]. Polyvinylpyrrolidone–iodine with slow release of iodine also provided this film with antiseptic and antibacterial properties. Combining the flexibility, oxygen permeability, high bacterial killing efficacy, biocompatibility, and conductivity (10 kΩ sq^−1^), the author suggested this film with promise as bandage for wound healing application. However, according to cell viability assay on human keratinocytes, the application of this film meets limitation. Iodine-containing films were suitable for severely infected wound but should be limited in the wounds that require rapid proliferation of keratinocytes and other mammalian cells.

Graphene possessing excellent conductivity at room temperature than any other carbon materials, a high optical absorptivity, high thermal conductivity, and high mechanical strength has great value in many fields [135]. However, graphene tends to aggregate, and the poor dispersion extremely restricts its application, especially as biomaterials. Graphene oxide (GO) and reduced graphene oxide (rGO) are covalently functionalized graphene which have been mostly studied [5, 53]. A large number of oxygen-containing groups as hydroxyl, epoxy, and carboxyl group supports GO with good dispersion. However, the covalent functionalization of carbon atom converting the planar *sp*^2^ hybridization to a tetrahedral *sp*^3^ hybridization would largely reduce the conductivity compared with graphene. For example, Shahmoradi et al. reported a graphene/silver incorporated wound dressing, only taking the advantages of reinforced mechanical properties of GO and its synergistic antibacterial effect with Ag NPs, without considering the electroactivity [136]. rGO containing few unreduced, covalently bonded oxy groups has a relative higher conductivity than GO [53]. Aycan et al. designed a conductive polymeric film consisting of sodium alginate, gelatin, hyaluronic acid and rGO [97]. The homogeneous rGO suspension was formed after ultrasonic treatment. rGO was incorporated into the polymeric network through strong Van der Waals interaction and hydrogen bonding. The conductive film demonstrated enhanced mechanical properties, good biocompatibility, adequate water vapor transmission rate, and improved oxygen permeability. Moreover, this conductive film was loaded with an anti-inflammatory drug ibuprofen and demonstrated a controlled release profile. Therefore, the author suggested this conductive film has great potential in wound dressing. Khamrai et al. synthesized a cellulose/rGO/Ag NPs composite film as multifunctional dressing with conductivity, and antimicrobial activity [117]. Notably, as the cellulose was modified with dopamine, this film exhibited an adhesive nature which could facilitate the adhesion and proliferation of NIH 3T3 fibroblast cell. A multifunctional composite film based on similar components was developed by Zhang et al. [121]. The difference is that polydopamine was utilized as a ligand to prevent Ag NPs agglomeration and silver loss.

#### Metals and Metal Oxides

Ag and Zn dots printed on polyester sheets or cotton nonwovens could work as bioelectronic wound dressings and have already proven significant effect on the improvement of human keratinocyte, cellular behavior activation, and wound healing by generating sustained microelectric potentials and restoring disrupted physiologic bioelectric signals on wound sites, while the voltage was depended on the size of the dots and the distances between metal dots [45, 111, 120].

**Fig. 4:**
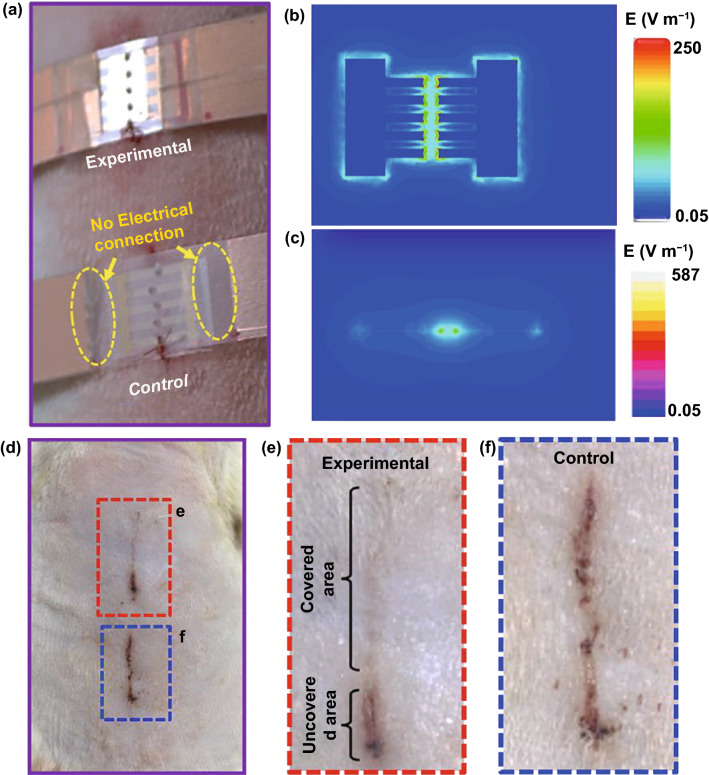
Liner wounds covered with dressing electrodes with or without electrical connection (**a**). Electrical field distribution on wound site from front (**b**) and lateral (**c**) view. Photographs (**e**) and correspondingly enlarged images (**f**) of wounds in different groups after treatment for 2 days. Reprinted from Ref. [116]. Copyright 2018, American Chemical Society

As mentioned above, the application of electrotherapy generally requires extracorporeal power supplies, which is not convenient for patients. Recently, the innovation of triboelectric nanogenerators that could harvest biomechanical energy and further generate periodic ES has expanded the development of facile electrotherapy [78, 137]. Zinc oxide nanorods can be integrated onto PDMS film assembling a highly bendable, stretchable piezoelectric dermal patch [115]. Since a piezoelectric potential was generated upon mechanical deformation induced by animal motion, the wound was under continuous stable ES. The patch displayed therapeutic effect to wounds and promoted cellular metabolism, migration, and protein synthesis. Fu et al. developed an efficient electrical bandage based on Cu foil as the electrodes, and polytetrafluoroethylene as the triboelectric active layer, and polyethylene terephthalate as the static substrate [116]. The bandage had good adaptability to soft skin. When the rat was equipped with a bandage around the chest, an alternating discrete electric field was generated from rat breathing. As shown in Fig. 4, liner wound covered with the bandage which connected with nanogenerator was under a strong and localized electrical field. Compared with the unclosed wound in control group, wound under electric field nearly fully closed after wearing the bandage for 2 days. From in vivo animal assay on large rectangular wounds, wounds covered with the bandage complete wound contraction within 3 days, much rapid than the wounds wearing the same device but without electrical connection.

#### Other 2D Inorganic Nanomaterials

**Fig. 5:**
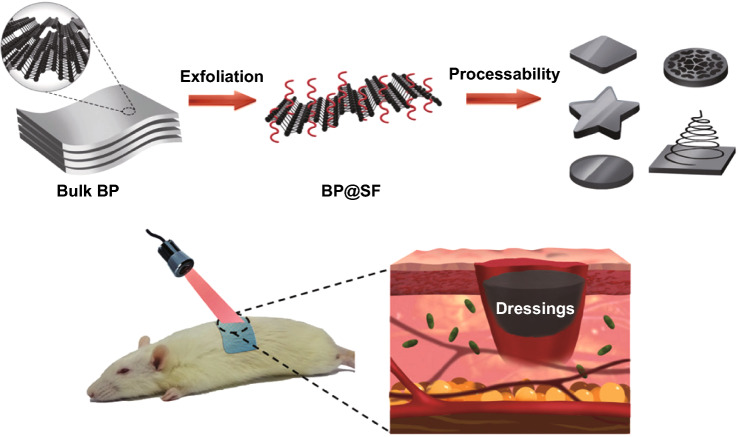
Schematic illustration of the fabrication of BP@SF possessing versatile solution-processability and its application for wound healing. Reprinted from Ref. [122]. Copyright 2018, American Chemical Society

Black phosphorus (BP) as a 2D layered semiconductor material demonstrates novel electrical and optical properties including excellent charge carrier mobility, tunable bandgap, and remarkable photothermal conversion efficiency [81]. Considering the excellent photothermal effect [91] and biocompatibility [90], BP has been regarded as a promising candidate for biomedical applications [91], such as cancer therapy [138], antibacterial therapy [139], and wound healing [94]. However, BP nanosheets are commonly synthesized via liquid exfoliation with organic solvents and could not stand a long-term usage because they would degrade rapidly under the physiological environment. In 2018, Huang et al. employed SF as the exfoliating agent to fabricate BP nanosheets via liquid exfoliation method in aqueous solutions as shown in Fig. 5 [122]. The BP hybrid nanosheets (BP@SF) demonstrated long-term stability, excellent photothermal antibacterial activities, and enhanced wound healing performance. Notably, due to the assistance of SF, BP can be easily fabricated into various forms, including fiber, film, and sponge, thus benefiting further applications in diverse types of wounds. BP can be more easily incorporated into hydrogels, nanofibers and other scaffolds by sonication or electrostatic absorption [81]. More importantly, due to remarkable photothermal effect, BP-based conductive biomaterials can be used for combinational photothermal therapy and wound healing [140].

Another superstar of 2D inorganic nanomaterials in biomedical field is MXene, which has been applied in biosensors, supercapacitors, batteries, catalysis, and therapeutics [93]. Similar to BP, stability under physiological conditions is one severe issue that restricts the biomedical application of MXene [95]. The application of MXene in wound healing is in the preliminary stage. Even though some reports have validated its positive performance in promoting wound healing [141–143], there is no report about MXene-based conductive film as wound dressing yet.

### Membrane

Membrane dressing is a semipermeable biomaterial similar as thin film, except that membrane has a certain degree of water absorption capacity, and thus can be used on wounds with moderate exudate, such as superficial wounds, frictional wounds, scratching wounds, and skin donor sites [144, 145]. Gharibi et al. developed an aniline tetramer embedded polyurethane/siloxane membrane as intelligent wound dressing [146]. The introduction of inorganic siloxane moieties endowed the hydrated membrane with enhanced mechanical properties, because hydrolysis and condensation of methoxysilane moieties would display a reinforcement effect on the polymeric network [147]. It is worth to mention that after being crosslinked by siloxane moieties, doped with camphorsulfonic acid and further solvent extraction/purification, the membrane should be immersed into distilled water maintaining in a hydrated state. Compared with commercial polyurethane-based dressing with compact structure, these membranes exhibited improved water absorption and higher oxygen and water vapor transmission rate. Furthermore, Ag NPs were incorporated via in situ reduction by the embedded oligoaniline, endowing the membranes with antibacterial activities and enhanced conductivity. Therefore, these membranes demonstrated promising radical scavenging property, perfect antimicrobial activity, and improved fibroblast cells viability and proliferation, all indicating great potential as wound dressing. In their follow-up work, a series of polyurethane/siloxane-based conductive dressing membranes composed of castor oil, ricinoleic methyl ester and aniline tetramer with conductivity, high biocompatibility, remarkable analgesic and anti-inflammatory effects, and promotion on epithelialization were developed [148]. The conductive membranes and nonconductive membranes all demonstrated significantly enhanced wound healing performance than sterile cotton gauze in terms of rapid wound closure, complete re-epithelization of the wound, enhanced vascularization, and collagen deposition.

Commercial membrane modified with conductive component is another simple approach to fabricate conductive membrane. Polyethylenimine conjugated PPy nanopigments can be coated on a PE-fiber-constructed membrane through dip coating method [149]. This conductive membrane demonstrated excellent photothermal convertible performance, antibacterial activities, warming preservation, and anti-vasoconstriction in vivo.

### Micro- and Nanofibers

Micro- and nanofibers have great potential in tissue engineering for their fibrillar architectural resemblance to ECM [26]. The porous structure and high specific surface area grant oxygen permeation, nutrient exchange, and management of relatively large exudate through absorbency [150]. In addition, the fibers are semipermeable, and the small-sized pores could restrain the penetration of pathogens. Commonly, micro- and nanofibers-based wound dressings are soft and compliant, thus can be easily applied on wound sites, and retain conformity under human movement [74]. Moreover, fibers could also be assembled into any desired shape to accommodate any shaped wounds. Table 2 presents a list of micro- and nanofibers as wound dressings.

**Table 2:** Micro- and nanofibers for wound healing applications

Components	Fabrication method	Conductivity	Electrical stimuli	Cell type	Animal model	Refs.
PANI/CSA/ PLCL	Electrospinning	0.0138 S cm^−1^	20 mA, 200 mA	NIH-3T3 fibroblasts, mouse skeletal muscle cells	N/A	[151]
3ABAPANI/PLA	Electrospinning	2.4–8.1 mS cm^−1^	N/A	COS-1 fibroblast cell	N/A	[152]
PPy/SF	Chemical polymerization, coating	N/A	N/A	Adult human mesenchymal stem cells, human fibroblasts	N/A	[153]
Graphene/CS/PVA	Electrospinning	N/A	N/A	N/A	I, IV	[154]
PPy/PET	Chemical polymerization	63.4 ± 0.9 kΩ sq^−1^	5 V, width 10 s, period 1200 s	Human skin fibroblasts	N/A	[155]
PEDOT/PLLA	Chemical polymerization	0.1 kΩ sq^−1^	50 mV mm^−1^, 6 h	Human dermal fibroblasts	N/A	[156]
PPy/PET	Chemical polymerization, coating	N/A	100 mV mm^−1^, width 10 s, period 1200 s, or width 300 s, period 600 s	Human dermal fibroblasts	N/A	[157]
PEDOT/PHBV	Electrospinning, chemical polymerization	1.45 ± 0.03 μS m^−1^	N/A	Human skin fibroblast	N/A	[158]
PANI/MWCNT/PNIPMA	Template-assisted polymerization/electrospinning	N/A	N/A	L929 mouse fibroblast cell	N/A	[159]
PPy-I/PLLA/PGA	Plasma polymerization	N/A	N/A	Human embryonic kidney cells, human primary dermal fibroblasts keratinocyte, and tenocytes	N/A	[160]
PPy/PET	Chemical polymerization	N/A	50 and 100 mV mm^−1^, width 300 s, period 600 s, or width 10 s, period 1200 s	Primary human dermal fibroblast	N/A	[42]
PPy or PANI/SF	Chemical polymerization, coating	2.2 × 10^–5^–1.6 × 10^–4^ S cm^−1^	N/A	Human keratinocyte cells	N/A	[161]
GO/PVA	Electrospinning	N/A	N/A	L929 mouse fibroblast cell	I	[162]
PANI/CSA/Chitin	Electrospinning	4.2 × 10^–4^ S cm^−1^	N/A	Human dermal fibroblasts	N/A	[163]
Mxene/CS	Electrospinning/glutaraldehyde crosslinking	N/A	N/A	N/A	N/A	[142]
GO/Ag NPs/PCL/Arginine	Electrospinning	N/A	N/A	L929 mouse fibroblast cell	I	[136]
PPy/Iodine/PVP	Electrospinning/coating via a plasma process	N/A	N/A	Human keratinocyte cells	N/A	[164]
PANI/PCL/QCS	Electrospinning	N/A	N/A	L929 mouse fibroblast cell	I	[165]
PPy/PEO/CS/Col	Electrospinning	59.564 × 10^–3^–164.274 × 10^–3^ S m^−1^	N/A	Human fibroblast cell	N/A	[166]
BP/PEG/PCL/Gel/doxorubicin	Electrospinning	N/A	N/A	Mouse embryonic fibroblast cells, normal skin cells	III	[140]
rGO/PAA	Electrospinning	N/A	N/A	N/A	V	[167]

IV: Full-thickness wound on rabbit. V: S. aureus-infected full-thickness wound

One approach to produce conductive fibers is to coat the fibers with conducting polymers. Wang et al. employed such a method to fabricate conductive PET microfibers [155]. Thin and uniform PPy was deposited on the surface of the commercial PET microfibers without blocking inter-fiber space. Under pulsed ES of 5 V, these conductive fabrics exhibited enough conductivity and stability within 24 h to promote fibroblast growth.

**Fig. 6:**
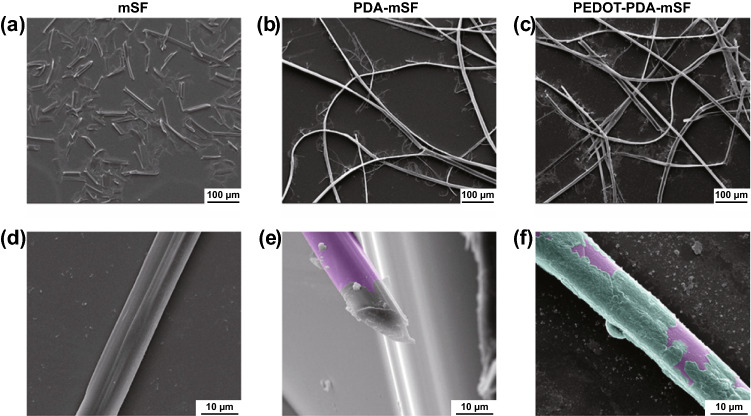
SEM images of SF microfibers extracted via traditional alkaline treatment (**a, b**), SF microfibers obtained with PDA protection (**c, d**), and SF microfibers coated with PDA and PEOOT (**e, f**). The fibers were colored with purple and green for clear observation of PDA and PEDOT layer. Reprinted from Ref. [168]. Copyright 2021, Wiley–VCH

Degummed silk fibroin (SF) is a natural protein fiber and has been extensively used as wound dressing [70]. GH et al. developed a simple strategy of in situ polymerization of pyrrole and aniline on SF fibers [161]. PPy and PANI were coated on SF by forming interactions with peptide linkages while mostly preserving protein assembly. Compared with the smooth surface of the primitive SF fibers, some particles and aggregates were found on the coated fibers, which would facilitate cell adhesion. As the conductivity of PPy/SF and PANI/SF fibers was at the same magnitude as the corresponding conjugated CPs, it suggested that this coating strategy was effective to construct homogenous and continuous conductive pathways on the surface of SF fibers. Compared with SF fibers, these two kinds of conductive SF fibers demonstrated positive effects on human immortalized keratinocytes adhesion and proliferation. Recently, Jia et al. reported conductive SF microfibers integrated bioelectronic with excellent conductivity for wound healing and diagnosis in diabetes. As shown in Fig. 6, the novelty of this work was the protection and pre-modification of SF microfibers with a PDA layer, which assisted the assembly and uniform deposition of PEDOT, as well as a well-connected conductive pathway.

However, this strategy is limited to polymers with a natural fibrillar structure or commercial fabrics products, and the diameter of these fibers cannot be adjusted. It is still of great necessity to develop fibers with tunable diameter ranging from nanometer to micrometer utilizing different polymers.

Electrospinning is a straightforward method to produce ultra-fine nanofibers or nanofiber assemblies with narrow diameter distribution [26]. Two general approaches have been developed to fabricate conductive electrospun nanofibers. The first one is the above-mentioned coating strategy. Cervantes et al. fabricated electrospun SF micro- and nanofibers with methanol treatment and then coated with PPy [153]. This PPy/SF conductive mat could promote human fibroblasts adhesion and proliferation. Besides, the diameter of SF fibers fabricated through electrospinning was much smaller compared with the SF fibers isolated from wild cocoons [161]. Due to its good solubility in water, PEDOT: PSS is suitable for this coating strategy. Chang et al. fabricated a polylactide and poly(3-hydroxybutyrate-co-3-hydroxyvalerate) electrospun membrane and then dipped this membrane in PEDOT: PSS solution. Notably, no change was found on the morphology of the membranes after coating treatment, while the wettability and surface roughness increased with the increase of PEDOT:PSS content that could facilitate cell adhesion, cell proliferation, and cell-scaffold interactions [158].

Another classic method to fabricate conductive fibers is direct electrospinning of conductive materials/polymer composites. The limiting factor may be the poor solubility or dispersion of these conductive materials. Zhang et al. managed to fabricate GO-modified electrospun polyvinyl alcohol nanofibers, and ultrasonication for 30 min was necessary for the homogenous dispersion of GO [162]. This conductive nanofibrous scaffold could promote growth and adhesion of L929 fibroblasts, because PVA supported excellent water absorption ability, biocompatibility, and physiological stability, and GO provided enhanced mechanical properties, electroactivity, and protein affinity. Graphene can be homogenously dispersed with PVA in DMF/H_2_O mixed solvent after ultrasound stirring treatment for 30 min. The designed nanofibers exhibited excellent antibacterial activities and enhanced wound healing effect [154].

**Fig. 7:**
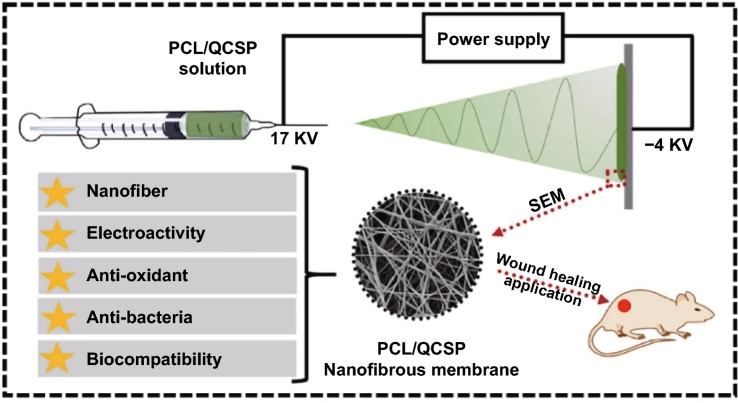
Schematic diagram of production and characteristics of PCL/QCSP NFMs. Reprinted from Ref. [165]. Copyright 2020, Elsevier

The insolubility of PANI in most solvents limits its application in direct electrospinning. Even though there are reports about fabricating PANI-incorporated nanofibers by direct electrospinning and validating their feasibility in skin tissue engineering, either a long-term pretreatment [163] or a low concentration of PANI solution was required [151]. Nikolaidis et al. synthesized a poly(aniline-co-3-aminobenzoic acid) copolymer with improved solubility in basic aqueous media and polar solvents which benefited further electrospinning [152]. Poly(aniline-co-3-aminobenzoic acid) and PLA blends could be well dissolved in dimethyl sulfoxide/tetrahydrofuran mixture. The corresponding nanofibrous mats doped with HCl have been confirmed as promising antimicrobial dressings owing to the conductivity, antimicrobial capability against Staphylococcus aureus, and enhanced COS-1 fibroblast proliferation. Recently, our group has developed a series of electroactive nanofibrous membranes (PCL/QCSP NFMs) based on quaternized chitosan grafted polyaniline (QCSP) and PCL, as illustrated in Fig. 7 [165]. In this membrane, QSCP segments provided multiple functions as excellent electrical conductivity, good solubility, antioxidant, and antibacterial activity, while PCL segments ensured the membranes with suitable mechanical properties. Results from in vitro cell culture and in vivo wound healing test and histological analysis have all confirmed that PCL/QCSP NFMs could promote cell proliferation, collagen deposition, and angiogenesis and accelerate wound healing process. Overall, conductive micro- and nanofibers can be fabricated through facile methods and promote wound healing effectively with good compliance to skin, easy removal, and suitable mechanical properties.

## 3D Conductive Biomaterials for Wound Healing

2D conductive biomaterials have made great achievement in wound healing. Through adopting multiple functions, they can promote cell attachment, proliferation, differentiation, and further the whole wound healing process. However, they are still restrained on thin and superficial wounds. In contrast, 3D biomaterials such as hydrogels, foams, and sponges possessing high water absorbance capacity can deal with wounds with high exudate [67]. On the other hand, deep wounds and chronic wounds have poor regenerative capacity due to versatile mechanism, such as lack of cell sources, severe infection, limited blood supply, immunosuppression or immunodeficiency, metabolic diseases, and other environmental factors [13]. Fortunately, 3D biomaterials possessing ECM-mimicking architecture could be utilized as scaffolds for these issues, which could not only support the integrity of dermis, but also act as carriers for bioactive reagents and cells [63].

### Hydrogel

Due to the porous 3D interconnected structure and high water content, hydrogel owns a lot of advantages as wound dressing [35, 145, 169]. Hydrogel allows oxygen and water vapor to pass through, maintains a humid environment, lowers the wound temperature, and relieves pain [170]. The soft nature, flexibility, and stretchability support hydrogel being compliant with human skin under ceaseless movement [171, 172]. Moreover, hydrogel has great tolerance to integrate multiple functions, including mechanical properties and additional therapeutic effects [173]. Correspondingly, hydrogel wound dressings have attracted most intensive attention in the past few years. Recently developed conductive hydrogel-based wound dressings are summarized in Table 3.

**Table 3:** Conductive hydrogels for wound healing applications

Application	Components	Crosslinking method	Conductivity (S m^−1^)	Electrical stimuli	Animal model	Refs.
Dressing	PANI/QCS/PEGS	Schiff base	0.225–0.35	N/A	I	[174]
Dressing	GO/Gel or Tyr/PAAm	Free radical polymerization	N/A	12, 24, 48 V	N/A	[175]
Dressing	PPy/PAAm	UV photopolymerization	0.3	0–900 mV	I	[176]
Dressing	BP/CS	Glutaraldehyde	N/A	N/A	V	[177]
Dressing	PPy/PEI/Gel	Ionic interaction, thermally reversible crosslinking	N/A	N/A	I	[178]
Dressing	rGO/HA/PDA	H_2_O_2_/HRP	0.5	N/A	I	[179]
Dressing	CNT/Gel/CS/PDA	H_2_O_2_/HRP	0.062–0.072	N/A	V	[180]
Dressing/electrode	PPy/PHEMA	UV photopolymerization	0.8	AC, 5 V, 40 Hz	III	[50]
Dressing	AT/CEC/OHA	Schiff base	0.9–4.2	N/A	I	[181]
Dressing	PANI/Ag NPs/PVA/PDA	Ionic interaction	N/A	N/A	VI	[182]
Dressing	CNT/CEC/PF127	Aromatic Schiff base	8.45 × 10^–3^	N/A	IX	[183]
Dressing	AT/HA	Click thiol-ene reaction	0.82	N/A	III	[184]
Dressing	rGO/PNIPAm/QCS/PDA	Covalent bond, Schiff base	0.5–0.56	N/A	I	[185]
Dressing	PEDOT/PSS/GS	Hydrogen bond, electrostatic interactions	0.22	N/A	I	[186]
Dressing	GO/Gel/CS	Covalent bond	2.29 × 10^–2^–10.07 × 10^–2^	N/A	VII	[187]
Dressing/electrode	MXene/Cellulose	Covalent bond, hydrogen bond, chain self-entanglements	2.83 × 10^–3^–7.04 × 10^–2^	100 mV mm^−1^, 1 h, every two days	I	[141]
Dressing	BP/fibrin/Lidocaine hydrochloride	Thrombin	N/A	N/A	III	[188]
Dressing	rGO/CS	Host–guest interaction	0.07–0.11	N/A	I	[189]
Dressing/electrode	PPy/Zn^2+^/CS/PVA	Di-diol complexation, hydrogen bonding, coordination bond	N/A	DC, 3 V	VIII	[190]
Microneedles	BP/PVA/Gel/Hemoglobin	UV photopolymerization	N/A	N/A	III	[191]
Dressing	Ag NWs/PVA/Agar/CEC	Boronate, hydrogen bond	N/A	N/A	IX	[192]
Dressing	Polycarboxybetaine	covalent bond	N/A	N/A	III	[193]
Dressing/electrode	LiCl/PAAm	UV photopolymerization	N/A	~ 10 Vpp	I	[194]
Dressing	MXene/PDA/HCHO	Schiff base	2.89 ± 0.025	N/A	III	[143]
Dressing	PAAm/PVA	Free radical polymerization	N/A	N/A	N/A	[195]
Dressing	Ag NPs/HTCC/OD-DA	Schiff base	N/A	N/A	V	[196]
Dressing	rGO/Tb^3+^/PVA/SA	Hydrogen bonding	N/A	N/A	VIII	[197]
Dressing	Ag–Graphene/PAA	Covalent bonding	N/A	N/A	I	[198]
Dressing	Ag NPs/SA/PNIPAm	Covalent bonding	N/A	N/A	I	[199]
Dressing/electrode	GO/Cellulose	Covalent bonding	> 6	≈300 mV	III	[200]

V: S. aureus-infected full-thickness wound. VI: Full-thickness diabetic foot wound. VII: MRSA-infected full-thickness wound. VIII: S. aureus-infected full-thickness diabetic wound. IX: S. aureus and E. coli-infected full-thickness diabetic foot wound

**Fig. 8:**
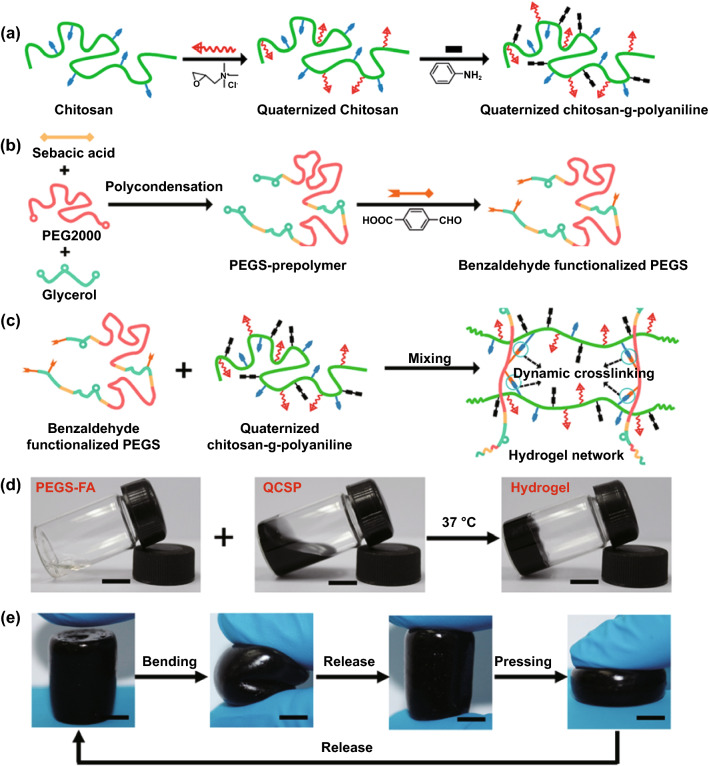
Schematic representation of QCSP/PEGS-FA hydrogel synthesis (**a-c**). Photographs of the gelation process (**d**) and flexible soft nature (**e**) of the hydrogel. Reprinted from Ref. [174]. Copyright 2017, Elsevier

So far, versatile conductive hydrogels with different conductive components have been developed. In contrast to most 2D biomaterials-based wound dressings which validate their potential in wound dressing by in vitro cell culture assay, the wound healing efficacy of most conductive hydrogels has been proved both by in vitro and in vivo animal assays. However, unlike the situations of film wound dressings where electrical activities of conductive substances can be isolated to prove their effects on cellular activities, the wound healing performance is always enhanced through synergistic effects from electrical activities, antioxidant, and antibacterial activities of hydrogel-based wound dressings. In 2015, Hsiao et al. synthesized a chitosan derivate with self-doped PANI which could form colloidal gels induced by pH increase and explored the photothermal antibacterial activities in vitro and on subcutaneous abscess [201]. But the conductivity and the electroactivity were not examined in this work. Since then, these relevant features of the conductive substances were comprehensively evaluated via standard protocols. Our group has designed multifunctional conductive hydrogel-based wound dressings and proven their positive performance in accelerating wound healing process [165, 179–181, 183, 185, 187]. Chitosan, a natural polysaccharide, has been utilized in wound dressing for a long time, owing to its inherent antibacterial, analgesic effect and hemostatic activity [202]. However, the poor solubility of chitosan under neutral and constrained antibacterial effectiveness under nonacidic environments extremely limit its application and efficacy of wound healing. Fortunately, the abundant active amino groups enable further modification. Quaternized chitosan (QCS) is a better choice for its excellent antibacterial activities and improved solubility. Our group not only solves the above issues, but also broadens its derivates and applications as wound dressing. As shown in Fig. 8, quaternized chitosan-g-polyaniline (QCSP) synthesized and demonstrated good water solubility and enhanced antibacterial activity and biocompatibility than pure chitosan [203]. Meanwhile, the residue active amino groups remained potential to react with other groups. In this work, QCSP was then crosslinked with benzaldehyde group functionalized poly(ethylene glycol)-co-poly(glycerol sebacate) via Schiff base forming the hydrogel network [174]. The optimal hydrogel dressing showed an ionic conductivity of 2.37 mS cm^−1^ that is close to that of human dermis, thus owning the ability to transfer bioelectrical signals for accelerating wound healing. Overall, compared with Tegaderm™ film, the optimal hydrogel dressing performed excellent enhanced wound healing covering all stages, including in vivo blood clotting capacity, promoted ECM synthesis, collagen deposition, granulation tissue thickness, and promoted remolding phase. We also developed a supramolecular conductive hydrogel based on QCS and graphene oxide-graft-cyclodextrin [189]. The dynamic host–guest interactions were employed as crosslinkers endowing the hydrogel with self-healing and injectability. Considering the antibacterial activity, cell proliferation, and hemocompatibility, the hydrogel with 0.4 wt% of rGO was selected as the optimal dressing. Indeed, this conductive hydrogel dressing exhibited enhanced wound healing on full-thickness wounds. It is worthy to mention that, we validated that Pluronic F127 and polydopamine are of great advantages in designing carbon nanomaterials incorporated hydrogels, including assisting homogeneous dispersion, improving mechanical properties and tissue adhesiveness [183, 185].

In addition, conductive hydrogels can promote the efficiency of electrotherapy. Commonly in clinic, small metal electrodes were attached on human body near the wounds; thus, ES could not directly cover the whole wound [194]. As the large impedance of human skin, it is only possible to apply ES on every inch of wounds under high voltage, which may threat more danger to the patients [24, 204]. In scientific research, conductive films and fabrics have been justified to promote wound healing under ES for covering the whole wound bed [42, 115, 155, 157]. Reasonably, conductive hydrogel is another good choice, in terms of conductivity, softness, stretchability, and flexibility. Recently, a conductive hydrogel containing poly(2-hydroxyethyl methacrylate) and PPy has justified superior to commercial hydrogel dressing considering the antibacterial capacity and alleviated secondary damage during removal. Moreover, the significance of this work was that the replacement of traditional separate electrodes with one integral conductive hydrogel can extremely promote the efficacy of electrotherapy [50]. Zhang et al. created a conductive hydrogel using Zn^2+^ and PPy as the conductive components and chitosan as the main polymer backbone. This hydrogel was capable of sensing temperature and strain variations and accelerating the infected chronic wounds with ES [190]. More impressively, Jeong et al. developed an ionic hydrogel dressing based on LiCl and combined the dressing with a prototypical wearable triboelectric nanogenerator [194]. The nanogenerator can harvest biophysical energy from friction between skin and deliver ES to hydrogel, while the hydrogel dressing directly distributes ES to the whole wound.

Another attractive feature of hydrogel biomaterial is the great potential in tissue engineering by acting as scaffold to support cells and biomolecules. Mesenchymal stem cell combined with an ECM-mimicking biomaterial has attracted much attention in chronic wound healing [205–207]. Conductive hydrogel has been employed as scaffold for the treatment of diabetic wounds. Jin et al. recently reported a conductive hydrogel scaffold based on AT, hyaluronic acid and gelatin [184]. Compared with nonconductive hydrogel, the conductive hydrogel was found to upregulate the level of Cx43, owing to better transport of molecules and ions between cells. What's even more impressive, O2-consuming enzyme laccase was introduced to cast a hypoxic microenvironment, and this hypoxic environment could maintain for almost 13 h. Furthermore, adipose-derived mesenchymal stem cells were loaded for direct delivery to the hostile wound, while relative cell activity remained higher than 85% within 2 days. Thus, this conductive hydrogel could act as a multifunctional scaffold for chronic wounds. Overall, conductive hydrogels could promote wound healing process via diversiform approaches, thus being regarded as valuable candidates for wound healing, particularly for complicated chronic wounds. On the other hand, the excellent conductivity, easy fabrication method, and facile surface modification enable conductive hydrogels with great potential in health care devices for wound diagnosis. But, the long-term durability of hydrogel may impede this progress.

### Fibrous Scaffold

Even though electrospun scaffolds have been reckoned as promising candidates for tissue engineering, their applications still constrained by several factors, such as pore size and pore interconnectivity that all affect cellular infiltration and tissue ingrowth into the scaffold. Small pore size did not hinder the application for nanofibers as wound dressing, but cell attachment and proliferation might be restrained on the surface of nanofibers [162, 164]. Nanofibers with too compact structures could not fulfill the requirements of porous scaffolds for tissue engineering applications [208]. A nonwoven conductive web composed of PEDOT and PLLA was fabricated by melt-spinning. After culture for 48 h, human dermal fibroblasts appeared throughout the scaffold, indicating the web permitted cell infiltration [156]. Another interesting work is about a polyaniline-multi-walled carbon nanotube/PNIPAm composite electrospun nanofibers-based “smart” scaffold with temperature responsiveness for cell delivery [159]. Above LCST, this conductive nanofibrous scaffolds demonstrated enhanced fibroblast attachment and proliferation, while below LCST, the encapsulated cells would detach and been delivered to human body. Moreover, this stimuli-responsive nanofiber network was inflammation-sensitive, and can deal with loco-regional acidosis, which helps to pass through the inflammation phase. Therefore, conductive nanofibrous scaffold could be envisioned with great value in skin tissue engineering with deliberate design.

### Sponge, Foam, and Acellular Dermal Matrix

Hydrogel-derived aerogel and cryogel have sponge-like structures and high polarity for water absorption and thus could not only manage with a large amount of water, but also permit water to flow out/in freely [209–213]. Foam also has an interconnected porous structure and is commonly manufactured from polyurethane or silicone [9, 15]. Without further modifications with a hydrophilic surface, the pure foam demonstrates hydrophobicity, thus benefiting inherent antibacterial properties [33]. The subtle difference between sponge and foam is that foam usually exhibited more enhanced mechanical properties than sponge [33]. Generally, foam and sponge dressings can be used for various types of wounds, including burn, ulcer, skin donor area, and transplant. Also, they are lightweight, elastic, and easy to use in practice. As the second layer of skin, the dermis consists of a connective ECM with fibroblasts, endothelial cells, smooth muscle cells, and mast cells [214, 215]. ECM supports the main structure of skin tissue, develops interactions with versatile growth factors, and modulates cellular activities [63]. Acellular dermal matrix (ADM) derived from human or animal skin has been widely used in tissue engineering and wound healing as tissue replacement, graft and wound dressing [216, 217]. The currently developed 3D conductive wound dressings are listed in Table 4.

**Table 4:** 3D Conductive porous biomaterials in wound dressing and skin tissue engineering

Type	Components	Fabrication method	Conductivity	Electrical stimuli	Animal model	Refs.
Foam	GO	Chemical vapor deposition	N/A	N/A	I	[205]
Aerogel	PPy/Ag/Cellulose	Freeze-drying, hydrogen bond	0.52 S m^−1^	N/A	N/A	[218]
Sponge	rGO/Isabgol	Freeze-drying, phosphate	N/A	N/A	I, III	[219]
Foam	PANI/PU/Usnic acid	In situ polymerization	20 MΩ cm	N/A	N/A	[220]
Sponge	BP/SF	Freeze-drying	N/A	N/A	II	[122]
Cryogel	CNT/PF127/CS	Freeze-drying, cryopolymerization	0.04—0.12 S m^−1^	N/A	N/A	[221]
Sponge	PPy/CNT/PU	Chemical polymerization	0.54 mS cm^−1^	N/A	N/A	[222]
Scaffold	rGO/ADM	EDC/NHS	N/A	N/A	III	[223]
Sponge	GO/PVA/SA	Freeze-drying	N/A	N/A	I	[224]
Scaffold	rGO/CS/SF	Freeze-drying, Schiff base, covalent bond	0.26 S m^−1^	300, 600, 900 mV	I	[225]
Foam	Ag NWs/PU	Spraying	24.5 Ω	100 mV mm^−1^	X	[226]

X: Full-thickness wound on pig

**Fig. 9:**
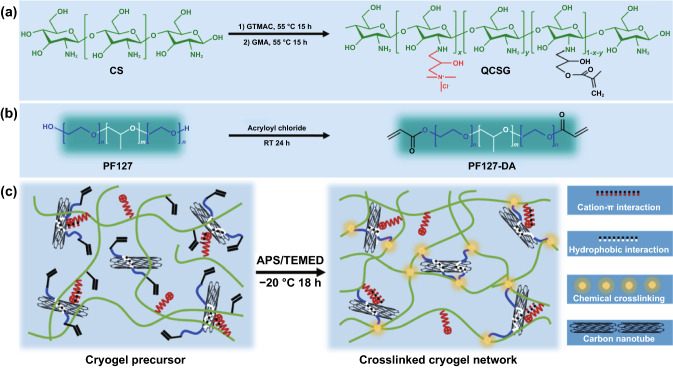
Schematic representation of QCSG/CNT cryogel synthesis (**a–c**). Reprinted from Ref. [221]

Our group developed a conductive cryogel composed of chitosan and PF127-assisted homodispersed CNT, while CNT providing conductivity and reinforcement toward the mechanical properties, as shown in Fig. 9 [221]. Compared with cryogel from pure natural polymers, commercial gelatin hemostatic sponge, and Combat Gauze, this CNT hybrid cryogel (QCSG/CNT) demonstrated rapid blood-triggered shape recovery and absorption speed, high blood uptake capacity, and hemostatic capability. Moreover, CNT provided this cryogel with photothermal effect and NIR-assisted photothermal antibacterial activity. Compared with commercial Tegaderm™ film and nonconductive cryogel, the conductive cryogel demonstrated better wound healing performance with the least inflammatory infiltration, and the highest vascularization by 15 days.

The ADM comprised of extracellular matrix proteins and collagen demonstrates excellent biocompatibility, suitable mechanical properties, and bioactivity which is ideal for skin tissue scaffold. Fu et al. developed a rGO-incorporated ADM-based scaffold via simple solution immersion process in which ADM was crosslinked with EDC and NHS [223]. The topology and structural integrity preserved after loading with rGO. Compared with the primitive scaffold and scaffold loaded with GO, ADM-rGO demonstrated superior cell attachment and proliferation for mesenchymal stem cells and human skin fibroblasts. Eventually, acting as a transplanting platform for mesenchymal stem cells, this conductive scaffold demonstrated enhanced therapeutic effect toward diabetic wounds. Based on the above, 3D conductive biomaterials have demonstrated encouraging results in promoting wound healing by working as wound dressings.

3D conductive biomaterials demonstrate promising potential in electrodes for electrotherapy and scaffolds for skin tissue engineering. Chen et al. reported an Ag nanowires-loaded foam demonstrating flexibility, enhanced conductivity, and long-term stability under physiological environment [226]. Due to the inherent antibacterial activity, good water-uptake capability, and electrical conductivity, the conductive foam could not only prevent infection and manage necrosis, but also implement annular oriented electrical field to wounds assisted by exogenous electrical fields. In the in vivo experiment on full-thickness pig skin wound, compared with control group treated with gauze, wounds treated with the conductive foam absent from exogenous electrical fields demonstrated enhanced wound healing performance for smaller wound residual area, controlled inflammation, better neovascularization, and advanced re-epithelialization. More excitingly, when applying the conductive foam with exogenous electrical fields, wounds demonstrated the most superior wound healing effect and therefore proved the great value of 3D conductive biomaterials in wound dressing, as well as their application in electrotherapy. Furthermore, the intrinsic feature of the highly porous structure enabled this conductive foam to connect with negative-pressure drainage closure device, thus simultaneously promote the wound healing process. Especially, since the structure, composition, appendages, and healing mechanism of porcine skin are closer to human skin, these results are more convincing.

## Application of Conductive Biomaterials in Wound Healing

**Fig. 10:**
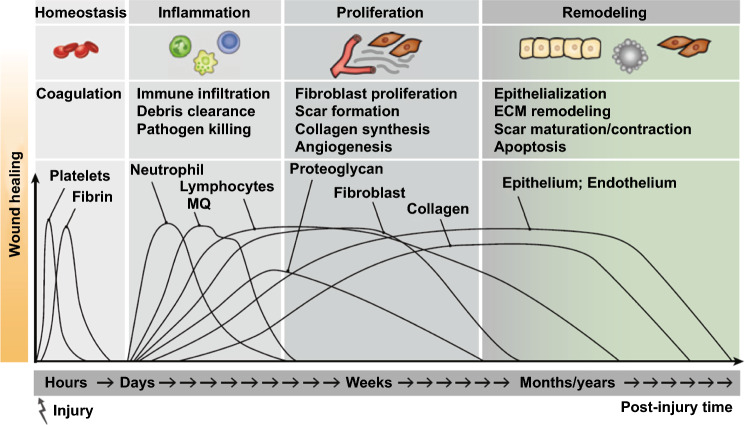
Sequential illustration of the four overlapping phases of classical wound healing. Reprinted from Ref. [227]. Copyright 2013, Elsevier

Regardless of different types of the wounds, the healing process occurs in a similar systematic manner including four distinct phases, as illustrated in Fig. 10 [227]. Ideally, hemostasis occurs immediately after injury and would complete within seconds or hours depending on wound size, depth, and wound location. Then, inflammation begins and lasts for several days and reaches the highest level by 72 h. The third phase, proliferation is more complicated. Angiogenesis, fibroblast migration, granulation tissue formation, collagen deposition, epithelialization, and wound contraction take place simultaneously. Finally, the last remodeling phase allowing granulation tissue to develop into mature connective tissue may last for several months to years. With standard wound care, acute wounds can progress through the healing routine steadily. However, in practice, normal wound healing would be affected and disrupted by many factors, including nutrition, oxygen supply, infection, aging, chronic disease, wound treatment, and even genetics. Extensive tissue damage, necrotic debris, and diseases often make wounds suffer from such issues, thus leading to prolonged inflammation and delayed proliferation and remodeling. Wound with delayed healing more than 3 months would be referred to as chronic wound [10, 228, 229]. In detail, chronic wounds with impaired regenerative capacity demonstrate high levels of proinflammatory cytokines, persistent infections, and drug resistance. Apparently, chronic wounds need specific treatment including tissue debridement, infection clearance, moisture balance, mechanical support, and management of comorbidities according to the etiology and real-time diagnosis.

Wound dressings and skin tissue scaffolds are of great importance in wound care and skin tissue regeneration [27, 160]. There have been developed plenty commercial products to fulfill the requirements for different wounds. Biomaterials with specific functions have also been symmetrically studied in promoting wound healing, such as antibacterial, hemostatic, adhesive, injectable, and antioxidant property. Conductive biomaterials demonstrate promising potential in wound healing as well, because they could regulate and promote cell attachment and relevant activities with or without ES that have been convinced by in vitro and in vivo assays [44, 61, 230]. So far, researchers have successfully validated the effectiveness of conductive biomaterials in different types of wounds, both for acute and chronic wounds. Moreover, due to the intrinsic electroconductivity, conductive wound dressings can be applied in real-time diagnosis. It is worth mentioning that conductive biomaterials are usually combined with other bioactive substances to meet the requirements in practice.

### Acute Wound

An acute wound is an unintentional injury to skin that can be caused by surgical incisions, bites, deep lacerations, abrasions, and burns [2, 3, 11, 33]. Acute wounds can spontaneously heal in an orderly routine even without any external intervention. In scientific research, clean incisional and excisional wounds with controlled area and facile surgery are frequently utilized to evaluate the effectiveness of wound dressings. Generally, a full-thickness wound with clear edge is created by surgical incision on the back of rat or pig. Full-thickness wound means a loss of all layers of the skin and great potential of the exposure of underlying tissues. Deep infection and fluid exudate affect the healing process, as well [15]. To address such issues, conductive biomaterials-based wound dressings integrating multiple functions are of high needs.

#### 2D Conductive Biomaterials for Acute Wound

As incisional and excisional wounds on rat or pig skin have light exudate, 2D biomaterial-based wound dressings can meet the requirements of wound care. Conductive film, membrane, and nanofibers have all realized their applications in acute full-thickness wounds. CPs and oligomers incorporated biomaterials have obtained great attention for their facile synthesis. In 2015, Gharibi et al. developed a series of polyurethane/siloxane-based conductive wound dressing containing aniline tetramer moieties [148]. These wound membranes displayed electroactivity, antimicrobial activity, and antioxidant ability which could promote fibroblast growth and proliferation. Besides, these CSA-doped membranes revealed comparable equilibrium water absorption value and water vapor transmission rate to some commercially available dressings, and suitable surface hydrophilicity to support cellular activity. Thus, the authors suggested these membranes could work as wound dressings for acute and chronic wounds, because the above three parameters are important to evaluate whether a product could maintain a moist environment for wounds. In an in vivo animal assay last for 20 days, the designed membranes exhibited accelerated wound healing than commercial cotton gauze. Our group synthesized a conductive polyurethane film, in which PCL provided mechanical properties, PEG contributed to surface wettability and AT supported electroactivity [118]. Through in vitro and in vivo assays, the conductive film with 12% AT content revealed improved cell adhesion and proliferation, and enhanced wound healing performance than nonelectroactive commercial dressing. Recently, our group also proved the viability of conductive nanofibers as wound dressing in practice. The electroactive nanofibers were electrospun from PCL and QCSP, thus demonstrating suitable mechanical properties, electroactivity and antibacterial properties [165]. The microporous structure can not only support cellular activities, but also guarantee the nanofibers to absorb exudate from wounds. The balance between antibacterial activity and cell proliferation should be taken into consideration as well, for bilateral properties of QCSP. Eventually, the conductive nanofibers with 15 wt% of QCSP were selected as the optimum dressings. Indeed, compared with Tegaderm^TM^ film, the electroactive nanofibers exhibited improved wound healing efficiency with rapid wound contraction, higher collagen deposition, lower production of TGF-α, and higher expression of VEGF within 14 days.

**Fig. 11:**
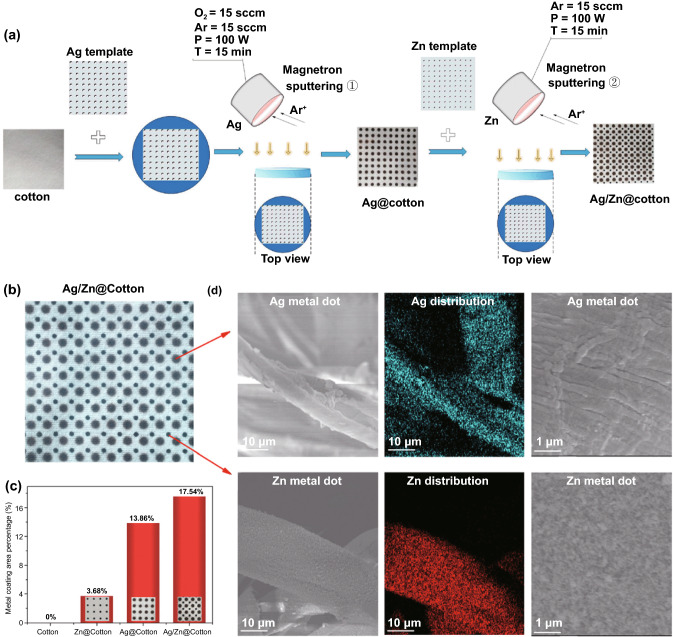
Schematic illustration (**a**), photograph (**b**) and metal coating area percentage (**c**) of Ag/Zn@Cotton dressing. (**d**) SEM images and corresponding EDS mapping of metal dots on the Ag/Zn@Cotton dressing. Reprinted from Ref. [120]. Copyright 2020, American Chemical Society

Except for the antibacterial activities, metals and metal oxides can generate ES under specific conditions. Liu et al. utilized template-assisted magnetron sputtering method to modify commercial spunlace cotton nonwovens with metal dots (Ag, Zn), as shown in Fig. 11 [120]. The low content of metals enabled the dressing with good cytocompatibility. Interestingly, this conductive Ag/Zn@Cotton dressing demonstrated enhanced cell migration and accelerated wound healing, which was attributed to the generation of ES and inherent antibacterial activities of Ag^+^/Zn^2+^ with continuous release under moist conditions. Bhang et al. developed a piezoelectric dermal patch based on zinc oxide nanorod and applied this patch in treating full-thickness wounds [115]. Under small mechanical deformations, this patch generated electrical fields. In animal assay, the patch was found to promote wound healing process via a series of cellular activities, including inflammation regulation, cell proliferation, re-epithelization, angiogenic factor secretion, and tissue remodeling.

In common, to address complications in acute wounds, 2D conductive biomaterials always need to be endowed with multiple bioactive functions while fulfilling basic requirements. However, the application of 2D conductive biomaterials in acute wound healing is still restricted by some parameters, such as the limited capacity for managing exudate, loading bioactive agents and maintaining their biological activities, further functionalization, and low adhesion to skin.

#### 3D Conductive Biomaterials for Acute Wound

3D biomaterials including hydrogels, foams, and sponges possess great potential in assembling ECM-like structure, so they have attracted much more attention in wound dressing and skin tissue scaffolds. Since there are diverse fabrication methods that can circumvent the limitations of these conductive substances, all types of conductive substances have been incorporated into various forms of 3D biomaterials and proved their merits in acute full-thickness wound treatment.

**Fig. 12:**
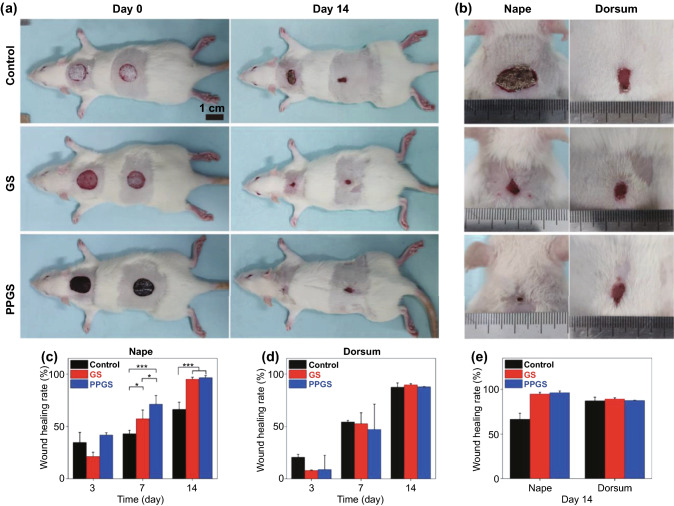
Wound healing in the stretchable parts of rats. Photographs (**a**) and correspondingly enlarged images (**b**) of wounds treated with different dressings. (**c–e**) statistics of wound healing rate on nape or dorsum by determined time. Reprinted from Ref. [186]. Copyright 2020, Wiley–VCH

Compared with 2D biomaterials, 3D biomaterials owning highly interconnected porous structure demonstrate several advantages. Bioactive agents including drugs and growth factors can be easily loaded into 3D biomaterials and exhibit sustained release profiles, which can benefit would healing [183, 185]. The higher water absorption capacity makes 3D biomaterials fit for wounds with large exudate and avoid frequent removal. The injectability and self-healing capacity at ambient environment make the hydrogel-based wound dressing suitable for irregular and deep wounds [171, 174, 181, 189]. Moreover, the mechanical properties of hydrogels could be easily adjusted to have suitable modulus and highly stretchable to comply with wounds at any part of the body, especially for wounds under large and incessant movement [171, 174, 179, 195]. Recently, Li et al. presented a conductive hydrogel based on PEDOT:PSS and guar slime, and verified its application on wounds in stretchable parts of the body [186]. The hydrogel exhibited rapid gelation within 1 min, injectability and self-healing ability. Compared with the dorsum of rats mostly being in static, nape is in frequent movement including compression, tension and twist. As shown in Fig. 12a, b, the designed dressings were applied on wounds constructed on the nape and dorsum of rats. Obviously, large movement would lead to delayed healing process. But when treated with a compliant conductive hydrogel-based wound dressing, wounds on the nape demonstrated an improved healing process according to the statistical data summarized in Fig. 12c–e. For this reason, 3D conductive biomaterials with compliance and high adhesiveness have paved way for the treating of wounds under incessant movement, for they could not adhere tightly to the wound without extra assistant, but also maintain structural integrity supporting full coverage for wound bed.

Due to the low adhesiveness, traditional wound dressings and novel 2D biomaterial-based wound dressings always require additional medical tape to be retained in wound sites. Large wounds often need commercial adhesives to promote wound closure and healing. Some novel adhesive conductive hydrogel-based wound dressings solve these two issues and improve the hemostatic effect at the same time. Conductive hydrogels containing Schiff base [174, 181] or polydopamine [179, 180] have been proven with high tissue adhesiveness, comparable or even better to that of commercial dressings. To combine the advantages of conductive biomaterials and adhesiveness, our group developed an injectable, self-healing hydrogel (QCS/rGO-PDA/PNIPAm), containing PDA and QCS for antibacterial properties and strong adhesiveness, PNIPAm for biomechanical activities, and rGO for electroconductivity [185]. Eventually, this hydrogel significantly promoted the full-thickness wound healing process demonstrating higher granulation tissue thickness, collagen disposition, and enhanced vascularization. The enhanced wound healing effect of this conductive hydrogel could be ascribed to accelerated wound closure through biomechanical adhesiveness and multiple biochemical functions simultaneously.

**Fig. 13:**
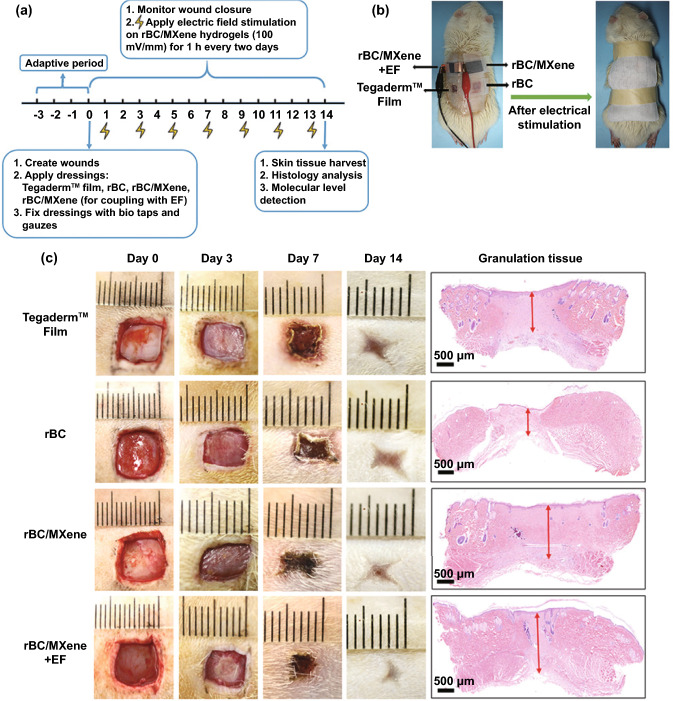
Schematic representation of in vivo wound healing experiment assisted by ES (**a**). Photographs of wounds treated with different wound dressings (**b**). Photographs of wounds on determined times and granulation tissue on day 14 in different groups (**c**). Reprinted from Ref. [141]. Copyright 2020, Wiley–VCH

Conductive hydrogels can also work as electrode to promote the efficiency of electrotherapy in curing full-thickness wounds. Mao et al. employed a regenerated bacterial cellulose/MXene composite hydrogel as the wound dressing and electrode for ES [141]. The composite hydrogel with 2% MXene content demonstrated the highest electrical conductivity, the best biocompatibility, and suitable mechanical properties. By in vitro electrostimulated cell culture assay and in vivo animal assay, this conductive hydrogel containing MXene was found to remarkably promote wound healing by applying 100 mV mm^−1^ DC electric field strength for 1 h every two days via wound contraction analysis and histopathologic evaluations, as illustrated in Fig. 13.

Overall, due to the versatile structures with high tolerance to accommodate multiple functions and properties, 3D conductive biomaterials have made great achievement in wound healing, especially for acute wounds.

### Chronic Wound

Chronic wound, including arterial, diabetic, pressure, and venous ulcers, is a serious threat to human health, and it takes decades to heal and accompanies by severe complication, amputations and even death [10, 33]. Traditional passive wound dressings are not effective enough for wound care of chronic wounds, because they could only provide protection against from exposure and moist balance [35, 67, 145]. The tissue debridement and infection control need further surgery and drug delivery. Overall, novel 3D conductive biomaterials integrating wound care and treatment have been paid much attention and need further development. Infected wound is one classic type of chronic wounds. Ideally, asepsis wounds will pass through the inflammatory phase after 2–5 days and gradually proceed into the proliferation and remodeling phases. Excessive and prolonged inflammation is obnoxious inevitably results in delayed healing and even death [231]. Actually, chronic wounds including diabetic wounds and ulcers could hardly proceed beyond the inflammatory phase [232]. Disinfection of infected wounds and prevention of wounds from bacteria invasion during the entire healing procedure are both essential for wound management [233]. Conductive biomaterials certainly exert positive effects during the inflammatory phase ascribing to their inherent antibacterial activities and photothermal antibacterial properties if necessary, thus prompting the transition to the proliferation phase. Besides, conductive biomaterials have been proved to exhibit antioxidant activity and enhance cell attachment, migration, and proliferation, which benefits both the inflammatory, proliferation, and remodeling phases [234]. In addition, when applied as electrodes in electrical therapy, conductive wound dressing can improve cell migration, alignment, proliferation and differentiation with specific programmed electrical stimulation [235]. In overall, conductive biomaterials could enhance wound healing through multiple pathways. Nevertheless, considering the complex in different wounds especially for chronic wounds, conductive biomaterials need to be tailored with multifunction or combined with other specific bioactive agents.

#### Infected Wound

Bacterial infection has long been a severe threat to human health. On one hand, they could induce many diseases and increase more complication during the treatment. On the other hand, bacterial resistance caused by abuse of antibiotics continues presenting significant burden on public health [54, 236, 237]. Wound infection is one of these tough issues [238]. Microorganisms can invade wounds and induce inflammation. Rapid colonization and the biofilms would prevent re-epithelization, and prolong wound healing process, and eventually lead to chronic bacterial-infected wounds [10]. Besides, another issue antibiotics suffering is that they could hardly penetrate biofilms, thus resulting in poor antibacterial efficiency [239]. Fortunately, conductive substances including CPs [201, 240], carbon nanomaterials [177, 180, 183], metals and metal oxides [123], MXene [143], and BP [177] exhibiting intrinsic antibacterial and photothermal antibacterial activities are all good alternatives for antibiotics, because they are less prone to induce bacterial resistance. Commonly, they can be solely incorporated into nonconductive polymeric matrix, exerting excellent bacterial killing effect and promotion toward infected wounds [143, 177, 201, 241]. Among various matrix materials, chitosan and its derivatives have been frequently selected for their synergistic intrinsic antibacterial effect. Chitosan derivatives as N-carboxyethyl chitosan and quaternized chitosan have also been combined with GO [187] or CNT [183] in designing conductive hydrogel-based wound dressings. These conductive dressings demonstrated multifunctional features and realized higher degrees of wound closure and skin regeneration within 14 days.

**Fig. 14:**
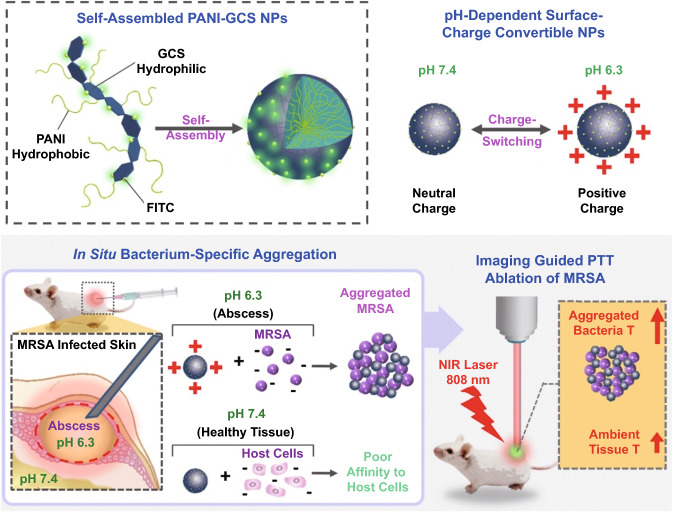
Schematic illustration self-assembly of PANI-GCS NPs in aqueous solution and the formation of bacteria and PANI-GCS NPs aggregates induced by acidity-triggered surface-charge conversion, thereby promoting photothermal ablation of focal infections. Reprinted from Ref. [240]. Copyright 2017, Elsevier

Conductive materials with nanostructure morphology owning increased membrane permeability and multiple antibacterial actions, are other preferential choices to deal with infected wounds [242]. In addition to the above-mentioned carbon-based nanomaterials that have been widely applied in infected wound management, nanometer-scaled conductive materials including CPs, metals and metal oxides, and semiconductors also show great value in promoted antibacterial efficiency. However, free nanomaterials are likely to be cleared rapidly from the interstices of tissues once being implanted owing to their small size [243]. Sung group reported chitosan derivatives containing self-doped polyaniline could self-assemble into nanostructures [240]. As shown in Fig. 14, polyaniline-conjugated glycol chitosan (PANI-GCS) could spontaneously form nanoparticles in aqueous solution. Since the surface-charge of PANI-GCS NPs was sensitive to surrounding environment, these PANI-GCS NPs suffered a bacterium-specific aggregation induced by localized skin infections which possessing acidic pus, while exerting no influence toward healthy tissues. By this method, the retention capability of PANI-GCS NPs at the injection area was significantly improved. Moreover, under NIR irradiation, there exhibited specific heating of PANI-GCS NPs/bacteria aggregates, the temperature dramatically reached 55 ℃, whereas a slight increase to 33 ℃ of the surrounding normal tissue. Presently, the encapsulation of conductive nanomaterials into multifunctional platform and combination with other bioactive agents have become necessary to implement their application in vivo.

Compared with antibacterial agents including Zn^2+^ and Cu^2+^ with narrow antibacterial spectrum, short-term durability, low heat resistance and stability, metal oxides as ZnO and CuO in nanostructure exhibit improved antibacterial capability, thereby possess great potential in curing infected wounds. Besides, due to the excellent antibacterial ability and extraordinary photothermal effect, Au NPs have excellent performance in killing bacteria. Wang et al. developed a polyvinyl alcohol composite film embedded with hybrid multi-shelled nanoparticles (ZnO@CuO@Au NPs) [123]. Under NIR laser irradiation, this composite film demonstrated enhanced ROS generation, destruction of bacterial cell membranes, and antibacterial efficacy, ascribing to the photothermal and photodynamic effect, and sustainably released Zn^2+^/Cu^2+^. Excitingly, MXene nanosheet as a novel class of 2D inorganic compounds of metal carbides and carbonatites with excellent conductivity, biocompatibility, and antibacterial ability, has shed light on the treatment of infected wounds, as reported by Zhang group [143]. Also, a BPs nanosheets-incorporated chitosan hydrogel has proved its effectiveness in treating S. aureus-infected skin wounds due to the production of singlet oxygen under simulated visible light, compared with pure chitosan hydrogel [177].

Combination of conductive materials and antibiotics is also a commonly used strategy for managing infected wounds. The synergistic effect from different antibacterial materials can not only reduce the drug resistance and ensure the antibacterial performance, but also alleviate the abuse of antibiotics. The drug-resistant methicillin-resistant *staphylococcus aureus* (MRSA)-infected wound model is well established to evaluate the efficiency of conductive biomaterials. Antibiotics, as doxycycline [180, 187] and moxifloxacin hydrochloride [183] with resistance toward MRSA have been combined with GO or CNT. The hydrogel matrices allowed for controlled and sustained release profile of antibiotics. Moreover, due to the efficient penetration of nanomaterials through biofilm, the antibiotic-loaded hybrid nanomaterials would largely increase the local concentration of antibiotics in the biofilm. Altinbasak et al. presented a rGO embedded PAA nanofiber mat, and antibiotics were simply loaded through immersion. This composite mat exhibited low passive diffusion-based release at ambient environment, whereas realized “on-demand” release tuned by power density of applied irradiation. Indeed, these hybrid nanofiber mats with photothermal assistance demonstrated the supreme wound healing capability of MRSA-infected wounds. Despite the promising achievement in infected wounds, the fact should not be overlooked that conductive biomaterials are usually synergistically combined with antibiotics and photothermal therapy. Moreover, whether conductive biomaterials are effective enough to severely infected wounds with biofilms still needs further investigation.

#### Diabetic Wound

**Fig. 15:**
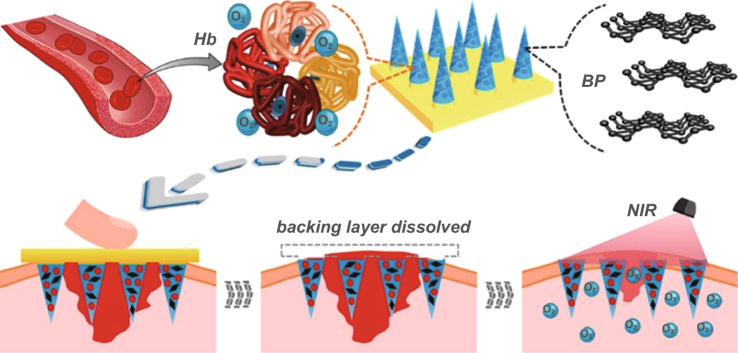
Schematic illustrations of thermo-responsive microneedles loaded with BP quantum dots and oxygen carrying hemoglobin. Reprinted from Ref. [191]. Copyright 2020, American Chemical Society

Compared with acute wounds, diabetic wounds exhibited prolonged infection, abnormal angiogenesis, and delayed re-epithelization. Therefore, the general principle of designing wound dressings and scaffolds for diabetic wounds is to prevent bacterial infection, control wound infection, induce angiogenesis, enhance collagen deposition, and promote cell proliferation [10, 74]. Even though conductive biomaterials have achieved excellent treatment effects on acute wounds and infected wounds, they are rarely used alone when dealing with diabetic wounds [182, 219]. Notably, conductive biomaterials encapsulated with insulin and fibroblast [244], or with mesenchymal stem cells have demonstrated enhanced diabetic wound healing performance [206, 207, 245]. Jin et al. presented an injectable conductive hydrogel based on aniline tetramer which could promote diabetic wound healing by incorporation of laccase to cast a hypoxic microenvironment maintaining for 13 h [184]. Such a hypoxia-pretreatment would largely enhance the effectiveness of adipose-derived mesenchymal stem cells when treating diabetic wounds. Subsequent adequate oxygen supply is essential for diabetic wound healing; thus, it is highly necessary to delivery oxygen to wound sites in a sustained and controlled manner. Zhang et al. developed BP contained thermo-responsive microneedles composing of polyvinyl acetate film as the backing layer and gelatin hydrogel loaded with BP quantum dots and hemoglobin as the tip, as shown in Fig. 15 [191]. Combining the photothermal effect of BP quantum dots and reversible oxygen binding property of hemoglobin, these microneedles realized NIR-controlled oxygen delivery. Under programmed intermitted NIR irradiation, these microneedles could support adequate oxygen supply lasted for 24 h. Indeed, these multifunctional microneedles demonstrated enhanced wound healing when treating full-thickness diabetic wound. On day 9, the group treated with BP quantum dots and NIR irradiation demonstrated most advanced healing performance, in terms of wound closure, granulation tissue width, epithelial thickness, and blood vessel density.

Electrotherapy has positive effects to treat chronic wounds but is still limited by small area of electrodes and uneven distribution of ES. Lu et al. have proved that applying conductive biomaterials as the electrodes in ES strategies could drastically improve the efficiency of electrotherapy [50]. Zhang et al. created a conductive self-healing hydrogel based on Zn^2+^and PPy [190]. The group of diabetic wounds covered with the conductive hydrogel and stimulated by a direct current voltage of 3 V for 1 h per day demonstrated the optimum wound healing performance than the group only covered with hydrogel and the control group. Overall, conductive biomaterials have demonstrated excellent performance in managing diabetic wounds though different pathways. Still, considering the variety and complexity of chronic wounds, the usage of conductive biomaterials, their combination with other reagents and specific implementation approaches need to be further explored [246].

### Wound Monitoring

Wound healing is a dynamic process comprising four overlapped stages, in which many parameters including humidity, temperature, pH, and glucose levels will change [247]. Compared with healthy skin, wounds demonstrate typical differences according to their types. On one hand, these differences could be utilized to design smart wound dressings that can specifically react to wounds but are inert to healthy skin [232, 239]. So far, numerous wound dressings with stimuli-responsiveness have been developed which can actively sense the variations and then self-adapt to the wounds. However, stimuli-responsive conductive wound dressings have not been widely explored [248]. At present, stimuli-responsive conductive wound dressings can be classified into two categories according to the sources of the two features. The first method is employing two distinct functional groups that endow wound dressings with stimuli-responsiveness and conductivity, correspondingly. Thus, the evaluation of stimuli-responsiveness and conductivity and their effects on wound healing could be studied separately. Zhao et al. reported a multifunctional hydrogel dressing consisting of boronate-based dynamic network and conductive component Ag NWs [192].The boronate-based dynamic network would collapse when treated with glucose, which benefits diabetic foot wound healing by facile on-demand removal. Eventually, wounds treated with this hydrogel dressing demonstrated rapid wound contraction rate and lower glucose level. Our group developed a hydrogel wound dressing exhibiting pH-responsiveness derived from Schiff base based network and conductivity from CNT [183]. The pH-responsiveness is conducive for controlled drug release when treating infected wounds which exhibiting acidic pH. In the second method, both stimuli-responsiveness and conductivity are derived from the same substance. Sung et al. synthesized a conductive chitosan derivate grafted with mercaptopropylsulfonic acid-doped polyaniline (NMPA-CS) and applied this derivate in treating infected wounds [201]. The CS derivative would self-assemble into micelles in acidic aqueous and form colloidal gel when increasing pH to 7.0. Thus, when injected into an infected wound, the NMPA-CS solution will completely cover the acidic area until gelation occurs when encountering healthy tissue. In their subsequent work, PANI-GCS NPs demonstrating positive charge under acidic environment can form aggregation with negatively charged bacteria, further facilitating photothermal ablation of focal infection [240]. Since the conductivity of CPs would be significantly affected by pH, the conductivity of the dressings would also change when the wound dressings undergo a specific change after sensing this stimulus [249].

On the other hand, wound monitoring has been developed. Diagnosis and monitoring of wounds are very imperative, especially for chronic wounds [6, 228]. Physical examination and the parameters including wound location, size, depth, and drainage should be well recorded and further treatment needs to be adjusted depending on the healing degree. However, long-term monitoring relies on patients’ hospitalization and frequent screening. Moreover, visual evaluation is far from accuracy and promptness. Some electrochemical sensors have been designed for wound diagnosis, but the wound dressing and wound treatment could not complete simultaneously [232, 250, 251]. Conductive wound dressings which can sense the wound variations and then convert them into electrical signals enabling synchronous wound care and wound monitoring are of great value in modern wound care. Recently, Zhao et al. fabricated an antibacterial conductive hydrogel as wound dressing based on polydopamine, Ag NPs, PANI, and PVA [182]. This hydrogel could directly adhere to human skin and respond to human mechanical deformation. Excitingly, they found that this hydrogel could distinguish diabetic rats from normal rats by movement, because diabetic rats have a relative slower respond to thermal stimuli. Jia et al. fabricated a PEDOT coated conductive silk microfiber integrated patch, and then employed this patch as ECG and EMG electrode for diagnosis in diabetes while promoting wound healing [168].

**Fig. 16:**
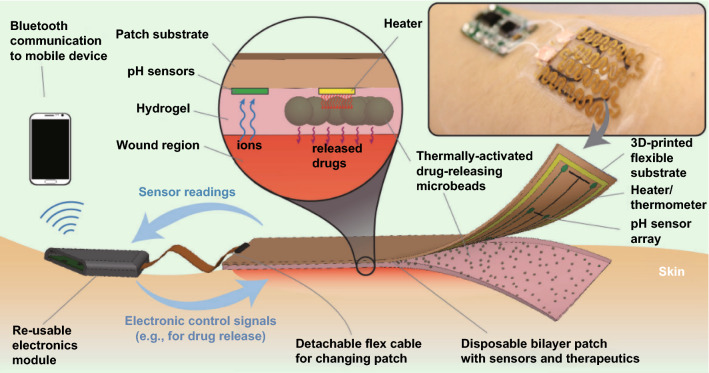
Schematic and conceptual view of the automated smart bandage for diabetic wounds integrating flexible pH sensors for real-time monitoring wound status and flexible heater for triggering thermo-responsive drug carriers loaded with antibiotics. Reprinted from Ref. [255]. Copyright 2018, Wiley–VCH

Another characteristic of chronic wound is the pH value. Compared with healthy skin with acidic pH ranging 5.5–6.5, chronic wounds exhibit alkaline pH between 7 and 9 or extremely acidic pH by severe infection [252, 253]. The level of pH can be continuously measured to monitor the chronic wound healing process. Recently, several works reported conductive biomaterials that can be used both as wound dressing and sensor. PANI is a proton-selective polymer and the conductivity of PANI is depended on protonation and deprotonation under different conditions [77, 82, 132]. Thus, PANI can be used to fabricate pH sensors monitoring wound status [254]. Mostafalu et al. developed a wound dressing integrating PANI-based pH sensors and flexible microheater with alginate hydrogel loaded with thermo-responsive drug carriers for antibacterial drug [255]. The dressing was also assembled with a wireless Bluetooth module for real-time monitoring, as illustrated in Fig. 16.

Glucose level is also a key factor of the diabetics. Lipani et al. reported a graphene-based thin film integrated with an electrochemical glucose sensor and proved this assembly platform could be applied as a noninvasive, transdermal glucose monitoring system to track blood sugar in human [250]. Thus, it could be anticipated with the emergence of wound dressing integrated real-time glucose sensing system in the future.

So far, real-time tracking of wound healing has been realized through monitoring the level of several parameters, including physiological signals [168], pH [193, 249, 251, 252, 254], oxygen [19], temperature [239], moisture [256], glucose [193, 250], and uric acid [257]. We envision that more comprehensive portable healthcare devices with high accuracy and precision based on conductive biomaterials will be manufactured, guaranteeing suitable wound care and noninvasive real-time healing measurement with comprehensive adaptivity at the same time.

## Summary and Perspectives

This review summarizes the application and achievement of conductive biomaterials in wound healing and skin tissue engineering. Conductive substances including CPs and their oligomers, carbon nanomaterials, metals and metal oxides, and novel 2D inorganic nanomaterials all have great advantages and serious drawbacks. CPs are limited by the poor processability, mechanical brittleness and nonbiodegradability, and the conductive oligomers benefit manufacture process and good biodegradability, but their conductivity under physiological conditions restricts further practical applications. Carbon nanomaterials and metals and metal oxides tend to aggregate in solution, and the homogeneous dispersion always requires aid from other polymers or techniques. The cytotoxicity of carbon nanomaterials and metals and metals oxides also matters their applications, especially in some researches that they are modified with CPs. More importantly, the conductivity of these materials dependeds on many factors, including pH value, dopant, and adjacent environment. But, in most articles, the measurement for the conductivity of biomaterials was taken place at specific conditions, which are totally different from the real conditions. BPs and MXene with great opportunities in wound healing are still facing a myriad of challenges before fulfilling application in wound healing.

Conductive biomaterials can be fabricated into diverse forms to meet the requirements of different types of wounds. 2D biomaterials as films, micro- and nanofibers, and membranes can treat acute wounds with fewer exudates, while 3D hydrogels and scaffolds with ECM-like structures are widely used in more complicated wounds and skin substitutes. Amidst, conductive thin films and hydrogel membranes can also work as substrates for bioelectronics, due to the soft feature, flexibility, and suitable mechanical properties [258–260]. Conductive film and micro-/nanofibers commonly possess much limited water-uptake ability, and the conductivity is always measured in the dry state. Therefore, they are suitable for wounds with low exudates. Conductive hydrogels and 3D porous scaffolds have high water-uptake ability, and the conductivity in dry and wet states both should be measured because of the ionic conductivity dominating in the wet state. Conductive hydrogels fit for wounds with moderate exudates, while 3D porous scaffolds can manage significant exudates. Moreover, conductive fibers, hydrogel, and 3D porous scaffolds can be loaded with drugs, growth factors, and cells, thus demonstrating great competitiveness in the multifunctional platform and even engineered skin substitutes.

Conductive biomaterials realize their applications in promoting wound healing via three strategies. First, they can be applied as compliant electrodes for electrotherapy. In general, the conductive wound dressings can facilitate ES to be well and uniformly conducted onto the wounds promoting the efficacy of electrotherapy [50, 109, 120, 225, 226]. Second, they can be used alone as wound dressings or tissue engineering scaffolds, demonstrating similar conductivity to human skin and supporting cellular activities, to accelerate wound healing performance. In addition, some conductive wound dressings loaded with bioactive agents have achieved controlled drug release assisted by an external circuit, realizing long-term treatment and lowering the initial burst and side effects [130, 131, 261–266]. In the absence of external ES, these conductive biomaterials still demonstrate enhanced cell attachment and proliferation, and promoted wound healing performance [37]. Also, the effectiveness of conductive biomaterials in promoting wound healing can be attributed to the inherent antibacterial and antioxidant capacities and photothermal properties of these conductive substances [77, 139, 149, 267]. Third, conductive biomaterials could be manufactured into stretchable and flexible electronics for real-time monitoring of wound status. With the significant progress and achievement of flexible electronics and wearable smart biomedical devices, scientists have managed to integrate conductive wound dressing with wound diagnosis and monitoring capacity [249, 255]. Such advance has extremely facilitated wound healing, because it can avoid frequent removal and replacement for closer observation. In contrast to visual and subjective observation, the instant automated evaluation with objective standards throws light on a more systematic analysis of wound healing and communication between patients and doctors.

In many cases, bioactive agents, including drugs, proteins, and growth factors have been incorporated into conductive biomaterials to enhance wound healing performance through versatile methods. The synergistic effects vary and should be discussed in the specific situation by considering the inherent properties of these bioactive agents, the interactions between bioactive agents and conductive materials or the polymeric matrixes, and the external conditions. Fibronectin as cell-adhesive glycoprotein was incorporated into PPy/PLLA film to endow the conductive film with enhanced fibroblasts adhesion and migration, while incorporation of BSA leading to reduced cell adhesion [106]. The analgesic and anti-inflammatory drug ibuprofen has been loaded into a PPy-based conductive film through electrochemical polymerization and realized on-demand, electronically controlled release under an electrical potential [130]. Curcumin, an effective drug demonstrating antioxidant, anti-inflammatory and antimicrobial activities, has long been limited by its hydrophobic nature [268]. Through the π − π interaction with aromatic ring in PANI, curcumin was entrapped within PHBV-g-PANI composite film [114]. This curcumin entrapped conductive film does not only reveal small burst release and controlled drug release profile due to the noncovalent interaction, but also demonstrate enhanced differentiation, proliferation, and migration of fibroblast cells ascribing to the incorporation of curcumin. Stem cells exhibited self-renewal ability and could differentiate into multiple types of cells, and their availability in promoting wound healing and skin tissue engineering has been widely validated [269]. Bone marrow derived mesenchymal stem cells have been loaded with a graphene foam [205]. The conductive scaffold was biocompatible and conducive for growth and proliferation of bone marrow derived mesenchymal stem cells. Eventually, the cell-loaded conductive foam was found upregulating vascular endothelial growth factor and basic fibroblast growth factor resulting in reduced scar formation in full-thickness defect experiment. The wound-specific delivery of growth factor is of great value in promoting wound healing efficiency, because the low concentration and excessive degradation of growth factor on wound site would delay the healing process [270]. Metal ions are of great value in maintaining human activities, wound healing process included. Copper ions and zinc ions have been incorporated into PEDOT-cellulose polymer composite through doping mechanism without affecting roughness topography of the substrate and realized controlled release. The combination of PEDOT with metal ions on cellulose substrate eventually contributed to enhanced attachment and proliferation of human keratinocytes [119]. Growth factors modulate a series of cellular activities for many types of cells, which is crucial in wound healing [64, 271]. Human keratinocytes’ directional migration under electric fields requires several growth factors, particularly epidermal growth factor [39]. In contrast to plenty of works about growth factors loaded conductive biomaterials for cardiac, muscle, and nerve tissue engineering [14, 270, 272, 273], the application of growth factors loaded conductive biomaterials in wound healing and skin tissue engineering has not been widely reported yet [274, 275]. Epidermal growth factor was loaded into a conductive polyacrylamide/chitosan hydrogel. Due to the coexistence of PPy nanorods and epidermal growth factor, the composite hydrogel demonstrated the optimal wound healing effects [176]. Based on the above facts, the combination of growth factors and conductive biomaterials is highly appreciated. The ways by which growth factors and conductive biomaterials combined, the interactions between them, and their application in specific types of wound healing need to be further explored.

In addition, the applications of conductive biomaterials are still in the preliminary stage, limited in acute wound, infected wound and diabetic wound. Whether conductive biomaterials wound promote the healing process for other types of wounds needs to be explored in the next, as well as the detailed mechanisms. Furthermore, there have not established some standard principles to compare the wound healing effects of these conductive biomaterials, as there are various animal models and different types of wounds. Even if in some works, the selected control groups as traditional passive wound dressings seem not convincing enough, because the chosen control group should be in the same morphology as the designed conductive biomaterials. Another issue is about the manufacturing process. The synthesis and incorporation of conductive substance are often complicated and sometimes require harsh conditions, which would restrict their further application and induce environment issues. Conductive substances are always combined with other functional materials. The interactions including synergistic effect or related side effects also should be fully evaluated.

So far, there are several commercially available health care products that contains silver ions, such as ACTICOAT antimicrobial silver dressing, AQUACEL® Ag foam, Biatain Silicone Ag, and SILVERCEL™ nonadherent dressing. But, these commercial dressings are mainly claimed for the antimicrobial capability. PosiFect RD® and Procellera® are wearable bioelectric dressings and have received FDA approval. They can provide electrical stimulation to wound. However, there are no commercially available conductive biomaterials based on other conductive agents for wound healing and skin tissue engineering. Since the application of conductive biomaterials as wound dressings for wound healing and skin tissue engineering is in the very preliminary stage, there are still many challenges for the further application in practice and clinic. Biocompatibility is one of the important criteria for biomaterials. In vitro short-term blood compatibility and cytotoxicity to different fibroblasts and keratinocytes are the most frequently used methods to evaluate their biocompatibility. However, there lacks the study of long-term histocompatibility of these conductive biomaterials. The biodegradation mechanisms of these conductive biomaterials under physiological environment are not all clear yet. The stability of MXene nanosheets and BP nanosheet under physiological environment is questionable. The conductivity of these conductive biomaterials is always measured under an ideal stable condition that is totally distinct from the real physiological environment. The real conductivity of these conductive biomaterials under practical conditions, how the conductivity would change along with the degradation and upon hydration or dehydration, whether the conductivity would surpass the safe range are still under question. Beyond that, surface modification is indispensable for majority conductive materials. However, it would significantly alter the properties of conductive nanomaterials, including conductivity, hydrophilicity, surface morphology, and photothermal effect, which are all crucial in wound healing. Even worse, metal and metal oxides, BP, and MXene lack of functional surface groups, which make this issue challenging. Moreover, almost all in vivo experiments were conducted on murine defects. But there exists great difference between human and murine skin. Even though the current research results on murine are encouraging, more systematic study and exploration must be conducted for the detailed mechanism in terms of each wound healing phase, while employing other large animal models to further verify the potential application in clinic.

In summary, the general method to fabricate conductive biomaterials is to incorporate small amount of conductive substance within other nonconducting polymers, and the properties of conductive biomaterials are mainly depended on the selection of the matrix polymers and crosslinking methods. Meanwhile, to accelerate the wound healing process in multiple channels, combination conductive biomaterials with other bioactive agents and cells is an effective method and needs more exploration. Moreover, with the development of nanogenerators and bioelectronics, electrotherapy and real-time wound assessment assisted by conductive biomaterials will make significant progress in the next decades. Working as wound dressing or electrode, conductive biomaterials have made significant achievement in wound healing, skin tissue regeneration and real-time wound diagnosis. Based on these achievements and the booming development of new technology, we expect that conductive biomaterials would make more advanced development for wound healing.

## Abbreviations

2D: Two-dimensional
3D: Three-dimensional
PSS: Poly(styrenesulfonate)
HA: Hyaluronic acid
PVS: Polyvinyl sulfate
Hep: Heparin
Derm: Dermatan sulfate
Fbri: Fibrinogen
Fn: Fibronectin
BSA: Bovine serum albumin
Tosylate: Toluenesulfonate
hDF: Primary human dermal fibroblasts
T98G: Human glioblastoma multiforme cells
APPY: Amine-functionalized polypyrrole
IL: Ionic liquid, 1-butyl-3-methylimidazolium tetra fluoroborate
RC: Regenerated cellulose
PHBV: Poly(3-hydroxybutyrate-co-3-hydroxyvalerate)
PDMS: Polydimethylsiloxane
PET: Polyethylene terephthalate
PTFE: Polytetrafluoroethylene
Gel: Gelatin
SA: Sodium alginate
PCL: Polycaprolactone
PEG: Polyethylene glycol
PCL-PEG-AT: Poly(polycaprolactone-co-polyethylene glycol-co-aniline trimer)
SF: Silk fibroin
a_m_: Amorphous zeolitic imidazolate frameworks
Ag NPs: Ag nanoparticles
PVA: Polyvinyl alcohol
PVPI: Polyvinylpyrrolidone-iodine
CSA: Camphorsulfonic acid
PLCL: Poly(L-lactide-co-ε-caprolactone)
3ABAPANI: Poly(aniline-co-3-aminobenzoic acid)
PLA: Poly(lactic acid)
CS: Chitosan
PNIPAm: Poly(n-isopropylacrylamide)
PGA: Poly(glycolic acid)
PLLA: Poly(l-lactic acid)
PPy-I: Pyrrole doped with iodine
QCS: Quaternized chitosan
Col: Collagen
PAA: Poly(acrylic acid)
PEGS: Poly(ethylene glycol)-co-poly(glycerol sebacate)
QCSP: Quaternized chitosan-g-polyaniline
Try: Trypsin
PAAm: Polyacrylamide
PEI: Polyethylenimine
PDA: Polydopamine
PHEMA: Poly(2-hydroxyethyl methacrylate)
CEC: N-carboxyethyl chitosan
OHA: Oxidized hyaluronic acid
PF127: Pluronic F127
GS: Guar slime
OD-DA: Oxidized dextran-dopamine
HTCC: Chitosan quaternary ammonium salt
HCHO: Oxidized hyaluronic acid
PU: Polyurethane
ADM: Acellular dermal matrix
ZnO NRs: Zinc oxide nanorods
EDC: 1-Ethyl-3-(3-(dimethylamino)propyl)carbodiimide hydrochloride
NHS: N-hydroxysuccinimide
ECG: Electrocardiogram
EMG: Electromyogram
TNF-α: Tumor necrosis factor-alpha
VEGF: Vascular endothelial growth factor
TGF-β1: Transforming growth factor-beta 1
α-SMA: Alpha-smooth muscle actin
TGF-β3: Transforming growth factor-beta 3
